# Scale length does matter: Recommendations for measurement invariance testing with categorical factor analysis and item response theory approaches

**DOI:** 10.3758/s13428-021-01690-7

**Published:** 2021-12-15

**Authors:** E. Damiano D’Urso, Kim De Roover, Jeroen K. Vermunt, Jesper Tijmstra

**Affiliations:** Department of Methodology and Statistics, School of Social and Behavioral Sciences, Tilburg University, PO Box 90153, 5000 LE Tilburg, The Netherlands

**Keywords:** Categorical data, Measurement invariance, DIF (differential item functioning), CFA (confirmatory factor analysis), IRT (item response theory)

## Abstract

In social sciences, the study of group differences concerning latent constructs is ubiquitous. These constructs are generally measured by means of scales composed of ordinal items. In order to compare these constructs across groups, one crucial requirement is that they are measured equivalently or, in technical jargon, that measurement invariance (MI) holds across the groups. This study compared the performance of scale- and item-level approaches based on multiple group categorical confirmatory factor analysis (MG-CCFA) and multiple group item response theory (MG-IRT) in testing MI with ordinal data. In general, the results of the simulation studies showed that MG-CCFA-based approaches outperformed MG-IRT-based approaches when testing MI at the scale level, whereas, at the item level, the best performing approach depends on the tested parameter (i.e., loadings or thresholds). That is, when testing loadings equivalence, the likelihood ratio test provided the best trade-off between true-positive rate and false-positive rate, whereas, when testing thresholds equivalence, the *χ*^2^ test outperformed the other testing strategies. In addition, the performance of MG-CCFA’s fit measures, such as RMSEA and CFI, seemed to depend largely on the length of the scale, especially when MI was tested at the item level. General caution is recommended when using these measures, especially when MI is tested for each item individually.

## Introduction

One of the main missions of psychological and social sciences is to study individuals as well as group differences with regard to latent constructs (e.g., extraversion). Such constructs are commonly measured by means of psychological scales in which subjects rate their level of agreement on various Likert-scale type of items by selecting one out of the possible response options. Most items’ response options range from 3 to 5 with a clear ordering (e.g., a score of 3 is higher than a score of 2 which is then higher than 1). Such items with few naturally ordered categories are called ordinal items.

Equivalence in the measurement of a psychological construct across groups is generally defined as measurement invariance (MI), and it is a crucial requirement to validly compare psychological constructs across groups (Borsboom, 2006; Meredith & Teresi, 2006). In fact, ignoring MI when statistically investigating differences between groups can lead to under/over estimation of group differences in item means (Jones & Gallo, 2002), sum-score means (Jeong & Lee, 2019), and regression parameters in structural equation models (Guenole & Brown, 2014).

In the context of psychological measurement, latent variable modeling is one of the most popular frameworks, and, within this framework, various approaches have been developed to model ordinal data as well as to test for MI. Among them, two of the most used ones are multiple group categorical confirmatory factor analysis (MG-CCFA) and multiple group item response theory (MG-IRT) (Kim & Yoon, 2011; Millsap, 2012). Interestingly, the difference between these two approaches is rather artificial, and parameters in MG-CCFA and MG-IRT models are known to be directly related (Takane & De Leeuw, 1987). Moreover, Chang et al., (2017) proposed a set of minimal identification constraints to make MG-CCFA and MG-IRT models fully equivalent.

The equivalence between these models, however, does not necessarily match the way MI is conceptualized and tested within each of the two approaches. For example, one main difference between MG-CCFA and MG-IRT refers to which hypotheses are tested. On the one hand, in MG-CCFA, measurement equivalence is mainly investigated at the scale level, or, in other words, the tested hypothesis is that the complete set of items functions equivalently across groups. On the other hand, in MG-IRT, more attention is dedicated towards the study of each individual item, and, for this reason, within this approach, MI is tested for each item in the scale separately. Another crucial difference relates to the way these hypotheses are tested. In fact, to test whether MI holds, either for a scale or for a specific item, different criteria and/or testing strategies are used within each approach.

Research to date has not yet determined the impact of these differences in terms of the performance to detect MI. For instance, some studies compared the performance of MG-CCFA and MG-IRT using solely an item-level testing perspective (Kim and Yoon, 2011; Chang et al., 2017), whereas Meade and Lautenschlager (2004) compared MG-IRT with multiple group confirmatory factor analysis for continuous data (i.e., MG-CFA). Providing clear guidelines on which approach to choose and in which setting is particularly helpful for applied researchers. In fact, having such guidelines might facilitate decisions regarding the level at which (non)invariance will be tested (e.g., scale or item level) as well as what are the most powerful tools to test it. However, in the current literature, clear guidelines have not yet been provided. Therefore, by means of two simulation studies, this paper makes three major contributions: (i) assess to what extent performing a scale- or an item-level test affects the power to detect MI, (ii) determine what MG-CCFA- or MG-IRT-based testing strategies/measures are more powerful to test MI, and (iii) based on the results of the simulation studies, provide guidelines on what approach to choose and in which conditions.

To this end, in “MG-CCFA, MG-IRT models and their MI test” we discuss both MG-CCFA- and MG-IRT-based models and illustrate how they are equivalent under a set of minimal identification constraints. Additionally, in the same section, for each of the two approaches, we discuss the differences in the set of hypotheses and the testing strategies in the context of MI. Afterwards, in “Simulation studies” we assess the performance of MG-CCFA- and MG-IRT-based testing strategies in testing MI by means of two simulation studies. Finally, in “Discussion”, we conclude by giving remarks and recommendations along with a summary of the main results obtained in the simulation studies.

## MG-CCFA, MG-IRT models and their MI test

### The models

Imagine having data composed of *J* items for a group of *N* subjects. Also, assume that a grouping variable exists such that subjects can be divided into *G* groups. Let *X*_*j*_ be the response on item *j* and further assume that *X*_*j*_ is a polytomously scored response which might take on *C* possible values, with *c* = {0,1,2,...,*C*-1}. Let us also assume that a unidimensional construct *η* underlies the observed responses (Chang et al., 2017).

#### Multiple group categorical confirmatory factor analysis

In MG-CCFA, it is assumed that *C* possible observed values are obtained from a discretization of a continuous unobserved response variable $X_{j}^{*}$ via some threshold parameters. The threshold $\tau _{j,c}^{(g)}$ indicates the dividing point for the categories (e.g., division between a score of 3 and 4). Additionally, these thresholds are created such that the first and the last one are defined as $\tau _{j,0}^{(g)}$ = -$\infty $ and $\tau _{j,c}^{(g)}$ = +$\infty $, respectively. Rewriting formally what we just described, we have:

1 $$ X_{j} = c,   if   \tau_{j,c}^{(g)} < X_{j}^{*} < \tau_{j,c+1}^{(g)}   \textit{c} =0, 1,2,...,C-1. $$

If it is also assumed that the construct under study is unidimensional, according to a factor analytical model we have:

2 $$ X_{j}^{*} = \lambda_{j}^{(g)}\eta + \epsilon_{j},   j = 1,2,...,J. $$

Equation (2) shows that the unobserved continuous response variable $X_{j}^{*}$ is determined by a latent variable score *η* via the factor loading $\lambda _{j}^{(g)}$ and a residual component *𝜖*_*j*_. The latter represents an error term that is item-specific. It is important to note that the thresholds $\tau _{j,c}^{(g)}$ and loadings $\lambda _{j}^{(g)}$ are group-specific. Additionally, both the latent variable *η* and the item-specific residual component *𝜖*_*j*_ are mutually independent and both normally distributed, with:

3 $$ {\eta^{(g)} \sim N(\kappa^{(g)}, \varphi^{(g)}),   \text{and}  \epsilon_{j}^{(g)} \sim N(0, \sigma_{j}^{2(g)}).} $$

where *κ* is the factor mean, *φ* the factor variance and ${\sigma _{j}^{2}}$ is the unique variance.

#### Multiple group normal ogive graded response model

MG-IRT models the probability of selecting a specific item category, given a score on the latent construct and given a specific group membership. These conditional probabilities, in the case of ordinal items, are modeled indirectly through building blocks that are constructed by means of specific functions. Different functions exist for ordinal items which, in turn, are used by different MG-IRT models. Because of its similarities with MG-CCFA (Chang et al., 2017), in the following, we only consider the multiple group normal ogive graded response model (MG-noGRM; Samejima, 1969). The MG-noGRM uses cumulative probabilities as its building blocks, and the underlying idea is to treat the multiple categories in a dichotomous fashion (Samejima, 1969). First, for each score, the probability of obtaining that score or higher is calculated (e.g., selecting 2 or above), given the latent construct *η*. Based on this set of probabilities, the probability of selecting a specific category (e.g., 2) is calculated, given a certain score on *η*. In the MG-noGRM, like in MG-CCFA, it is assumed that the observed values *X*_*j*_ arise from an underlying continuous latent response variable $X_{j}^{*}$.

Rewriting formally what we just described, the probability of scoring a certain category *c* is then:

4 $$ \begin{aligned} & P(X_{j}^{*} = c|\eta, g)\\ &= {\Phi}(\alpha_{j}^{(g)}(\eta- \delta_{j, c}^{(g)})) - {\Phi}(\alpha_{j}^{(g)}(\eta-\delta_{j,c+1}^{(g)}))\\ & = {\Phi}(\alpha_{j}^{(g)}\eta- \alpha_{j}^{(g)}\delta_{j, c}^{(g)}) - {\Phi}(\alpha_{j}^{(g)}\eta-\alpha_{j}^{(g)}\delta_{j, c+1}^{(g)}) \\ &= \displaystyle{\int}_{\alpha_{j}^{(g)}\eta - \alpha_{j}^{(g)}\delta_{j,c+1}^{(g)}}^{\alpha_{j}^{(g)}\eta - \alpha_{j}^{(g)}\delta_{j, c}^{(g)}} \phi(u_{j})du_{j} \end{aligned} $$

where, for group *g* $\alpha _{j}^{(g)}$ is the discrimination parameter for item *j*, and $\delta _{j, c}^{(g)}$ is the threshold parameter. The latter represents the point at which the probability of answering at or above category *c* is .5 for group *g*. Since ordered categories are modeled, the probability of getting at least the lowest score is 1, and the first threshold $\delta _{j,0}^{(g)}$ is not estimated and set to -$\infty $. That is, *C*-1 threshold parameters per group need to be estimated. It is relevant to highlight that, like in MG-CCFA, also in the case of the MG-noGRM the model parameters $\alpha _{j}^{(g)}$ and $\delta _{j, c}^{(g)}$ are group-specific. Also, *ϕ*(.) is the probability density function and Φ(.) is the cumulative distribution function of the standard normal distribution.

##### Similarities with MG-CCFA

The similarities between MG-CCFA and the MG-noGRM can be revealed by taking a closer look at how the parameters in the two models are related (Takane & De Leeuw, 1987; Kamata & Bauer, 2008; Chang et al., 2017):

5 $$ \alpha_{j}^{(g)} = \frac{\lambda_{j}^{(g)}}{\sigma_{j}},      u_{j} = \frac{\epsilon_{j}}{\sigma_{j}},      \delta_{j, c}^{(g)} = \frac{\tau_{j,c}^{(g)}}{\lambda_{j}^{(g)}}, $$

and how it is possible to write the probability of $X_{j}^{*}$ given *η* in MG-CCFA terms:

6 $$ \begin{aligned} & P(X_{j}^{*} = c|\eta, g) = \displaystyle{\int}_{\lambda_{j}^{(g)}\eta - \tau_{j,c+1}^{(g)}}^{\lambda_{j}^{(g)}\eta - \tau_{j,c}^{(g)}} \phi(\epsilon_{j})d\epsilon_{j}\\ &                   = \displaystyle{\int}_{\lambda_{j}^{(g)}\eta/ \sigma_{j} - \tau_{j,c+1}^{(g)}/ \sigma_{j}}^{ \lambda_{j}^{(g)}\eta/ \sigma_{j} - \tau_{j,c}^{(g)}/ \sigma_{j}} \phi(u_{j})du_{j}. \end{aligned} $$

The difference between (4) and (6) is that in MG-CCFA the loadings $\lambda _{j}^{(g)}$ and the thresholds $\tau _{j,c}^{(g)}$ can be inferred only in a relative sense. In fact, they can only be calculated through the ratio with the residual variance *σ*_*j*_ (Takane & De Leeuw, 1987; Kamata & Bauer, 2008; Chang et al., 2017). This is due to the absence of a scale for the latent response variable $X_{j}^{*}$. For ease of reading, in the following, only the term loading will be used to refer to both the discrimination parameters and the loadings.

#### Identification constraints and models equivalence

Identification of measurement models such as the ones considered here can be achieved by means of identification constraints, which are usually imposed either via specification of an arbitrary value for some parameters or by setting equalities across them. This way, the number of parameters to be estimated is reduced, and it is possible to find a unique solution in the estimation process (Millsap & Yun-Tein, 2004; San, 2013; Chang et al., 2017).

In testing MI with multiple groups, both for MG-CCFA and the MG-noGRM, it is necessary to ensure that a scale is set for (i) the latent response variable $X_{j}^{*}$, (ii) the latent construct *η*, and that (iii) the scale of the latent construct is aligned across groups such that the parameters can be directly compared (Kamata & Bauer, 2008; Chang et al., 2017). Interestingly, these constraints are commonly imposed in a different way in MG-CCFA and in the MG-noGRM.

The observed response for each item is assumed to arise, in both models, from an unobserved continuous response variable $X_{j}^{*}$. These underlying continuous response variables do not have a scale. For this reason, a scale has to be set by constraining their variances and means. In both models, the means of the latent response variables are indirectly constrained to be 0 by setting the intercepts *κ* to be 0, since $E(X^{*}_{j})$ = *λ*_*j*_*κ*.

In both models, the means of the latent response variables are constrained to be 0. However, different ways to constrain the variances are generally used. It is common to either set their total variances $V(X_{j}^{*})$ to 1 (also called Delta parameterization; Muthén, 1998) or its unique variances ${\sigma _{j}^{2}}$ to 1 (also called Theta parameterization; Muthén, 1998). The former is much more common in MG-CCFA, while the latter is closer to what is usually done with the MG-noGRM (Kamata & Bauer, 2008).

The other unobserved element for which a scale has to be set is the latent construct *η*. Again, this is commonly addressed in a different way in the two approaches. On the one hand, in MG-CCFA a fixed value is commonly chosen for a threshold and a loading. On the other hand, in the MG-noGRM the scale of the latent variable is commonly defined by setting its mean and variance to 0 and 1, respectively. In both cases, these constraints are applied only for one of the two groups, which is usually called the reference group.

Finally, it is necessary to align the scale of both groups to make them comparable. This is commonly achieved by imposing equality constraints on some of the parameters in the model, which is again addressed differently in MG-CCFA and in the MG-noGRM. On the one hand, in MG-CCFA for each latent construct, the factor loading and the threshold of a single item are constrained to be equal across groups. Generally, the loading and the threshold of the first item of the scale are selected. On the other hand, in MG-IRT, multiple items, assumed to function equivalently in both groups, are set equal by constraining their parameters. These items form what is then called the anchor. Note that, in the MG-noGRM, and more generally in MG-IRT models, greater attention is devoted to choosing the items that are constrained to be equal across groups while in MG-CCFA this is not necessarily the case. Nevertheless, in MG-CCFA, French and Finch (2008) have noted that the referent indicator matters, and various methods have been developed to select one or more referent indicators (Lopez Rivas et al., 2009; Woods, 2009; Meade & Wright, 2012; Shi et al., 2017). For a recent overview and comparison of these methods, we refer the reader to Thompson et al., (2021).

A set of minimal constraints to make MG-CCFA and the MG-noGRM fully comparable have been recently proposed by Chang et al., (2017), which will also be presented here. Without loss of generality, imagine that two groups, *g = r,f* where *r* represents the reference group and *f* the focal group, exist. Following Chang et al., (2017):

7 $$ \sigma_{j}^{2(r)} = 1,   \text{for \textit{j}= 1,..,\textit{J}} $$

8 $$ E(\eta^{(r)}) = 0,   {\lambda_{1}^{(r)} = 1}, $$

9 $$ \begin{array}{@{}rcl@{}} \lambda_{1}^{(r)} &=& \lambda_{1}^{(f)},   \sigma_{1}^{2(r)} = \sigma_{1}^{2(f)},   \tau_{1,c}^{(r)} = \tau_{1,c}^{(f)}, \\&& \text{for some \textit{c} $\in$ (0,1,2,...,\textit{C}-1)} \end{array} $$

10 $$ \sigma_{j}^{2(r)} = \sigma_{j}^{2(f)} \text{for \textit{j} = 2,..,\textit{J}}. $$

These constraints serve the purpose to set a scale for the latent response variable $X_{j}^{*}$, for the latent construct *η* and to make the scale comparable across groups. That is, (7) and (8) set the scales of the latent response variable $X_{j}^{*}$ and the latent construct *η* for the reference group, while (9) makes the scale comparable across groups using the anchor. Finally, (10) guarantees a common scale across all the other items. Furthermore, the above-mentioned constraints can be seen as MG-IRT-type constraints where the unique variances ${\sigma _{j}^{2}}$ are constrained to be 1 both for the focal and the reference group, the mean of the latent construct *η* is set to 0 and at least one item is picked as the anchor item, which parameters are set to be equal across groups (Chang et al., 2017).

By means of these constraints, the two models are exactly the same. Thus, differences in testing MI between MG-CCFA and the MG-noGRM depend only on the level at which MI is tested (i.e., scale or item) as well as what measures and testing strategies are used to test it.

### MI hypotheses

Generally, a measure is said to be invariant if the score that a person obtains on a scale does not depend on his/her belonging to a specific group but only on the underlying psychological construct. Formally, assume that a vector of scores on some items **X** is observed, where **X** {= *X*_1_, *X*_2_,..., *X*_*j*_}, and that a vector of scores on some latent variables *η* underlies these scores, where *η* {= *η*_1_, *η*_2_,...,*η*_*r*_}. Then, measurement invariance holds if:

11 $$ P(\mathbf{X}|\text{\boldmath$\eta$}, \mathbf{g}) = P(\mathbf{X}|\text{\boldmath$\eta$}). $$

Equation (11) shows that the probability of observing a set of scores **X** given the underlying latent construct (***η***) is the same across all groups. Moreover, the equation is quite general in the sense that no particular model is yet specified for *P*(**X**|***η***).

As discussed above, an equivalent model for *P*(**X**|***η***) can be specified for MG-CCFA and the MG-noGRM. Then, one of the main differences in the way these two approaches test MI is whether a test is conducted for the whole vector of scores at once or for each element of the vector separately. Although, in principle, both types of test can be conducted within each approach, the former is more common in MG-CCFA, while the latter is generally used within MG-IRT. However, in principle, both types of test can be conducted within each framework.

#### Scale level

In MG-CCFA, MI is tested for all items at once. Different model parameters can be responsible for measurement non-invariance, and they are tested in a step-wise fashion. In each step, a new model is estimated, with additional constraints imposed on certain parameters (e.g., loadings) to test their invariance. Then, the fit of the model to the data is evaluated to test whether these new constraints worsen it significantly. The latter being true indicates that at least some of the constrained parameters are non-invariant.

##### Configural

The starting point in MG-CCFA is testing configural invariance. In this first step, the aim is to test whether, across groups, the same number of factors hold and that each factor is measured by the same items. This is generally done by first specifying and then estimating the same model for all groups. Afterwards, fit measures are examined to determine whether the hypothesis of the same model underlying all groups is rejected or not.

##### Metric

If the hypothesis of configural invariance is not rejected, the next step is to test the equivalence of factor loadings. This step is also called the weak or metric invariance step. Commonly, the factor loadings of all items are constrained to be equal across groups. The hypothesis being tested here is that:

12 $$ H_{metric}: {\Lambda}^{(g)} = {\Lambda}. $$

If (12) is supported, the equivalence of factor loadings indicates that each measured variable contributes to each latent construct to a similar extent across groups (Putnick and Bornstein, 2016).

##### Scalar

If metric invariance holds, scalar invariance or invariance of the intercepts can be tested. In MG-CCFA, though, the observed data are assumed to come from an underlying continuous response variable $X_{j}^{*}$. This variable does not have a scale and, generally, its intercept is fixed to 0. That is why instead of the intercepts the thresholds are tested. To test the hypothesis of equal thresholds, these parameters are constrained to be equal across groups, while keeping the previous constraints in place. Formally, the hypothesis being tested is:

13 $$ H_{scalar}: T_{j}^{(g)} = T_{j}   \text{for \textit{j} = 1, 2, .., \textit{J}}. $$

If the hypothesis in (13) is not rejected, it can be concluded that the thresholds parameters for all items are the same across groups. Finally, it is worth noting that, to obtain full factorial invariance, equivalence of the residual variances should also be tested (Meredith & Teresi, 2006). However, many researchers do not consider this step, since it is not relevant when comparing the mean of the latent constructs across groups (Vandenberg & Lance, 2000).

#### Item level

In MG-IRT, the functioning of each item is tested separately. An item shows differential item functioning (DIF) if the probability of selecting a certain category on that item differs across two groups, given the same score on the latent construct. It is important to highlight that, when DIF is tested following a typical MG-IRT-based approach, configural invariance is generally assumed. Also, compared to MG-CCFA where item parameters are firstly allowed to differ and then constrained to be equal across groups, testing DIF follows a different rationale. That is, the starting assumption is that all items function equivalently across groups. Formally:

14 $$ \begin{aligned} & H_{0}: \alpha_{j}^{(g)} = \alpha_{j} = \frac{\lambda_{j}^{(g)}}{\sigma_{j}} = \frac{\lambda_{j}}{\sigma_{j}}, \delta_{j, c}^{(g)} = \delta_{j, c} = \frac{\tau_{j, c}^{(g)}}{\lambda_{j}^{(g)}} = \frac{\tau_{j, c}}{\lambda_{j}}\\ & \text{for \textit{j} = 1,2,..,\textit{J}, \textit{c} = 0,1,2,...,\textit{C}-1}. \end{aligned} $$

The constraints on one item are then freed up to test whether its parameters are invariant, while keeping the other items constrained to be equal across groups. Afterwards, the procedure is iteratively repeated for all the other items in the scale. DIF can take two different forms: uniform and nonuniform.

##### Uniform DIF

Given two groups, an ordinal item shows uniform DIF when, between groups, the thresholds parameters differ. In formal terms:

15 $$ \begin{aligned} & H_{no  uniformDIF}: \delta_{J/k, c}^{(g)} = \delta_{J/k, c} = \frac{\tau_{J/k, c}^{(g)}}{\lambda_{J/k}^{(g)}} = \frac{\tau_{J/k, c}}{\lambda_{J/k}}\\ & \text{for \textit{j} = 1,2,..,\textit{J}, \textit{c} = 0,1,2,...,\textit{C}-1 and for some \textit{k},}\\& \text{where \textit{k} = 1,2,...,\textit{J}}. \end{aligned} $$

where the subscript *J*/*k* stands for all items except item *k*. Equation (15) shows the hypothesis of no uniform DIF indicating that the thresholds of all items except item *k* (*τ*_*J*/*k*,*c*_) are the same across groups. Furthermore, it is interesting to note the connection between uniform DIF and scalar invariance, since both can be seen as tests for shifts in the threshold parameters.

##### Nonuniform DIF

An ordinal item shows nonuniform DIF when the loading parameter differ across two groups. The tested hypothesis can be formally written as:

16 $$ \begin{aligned} & H_{no nonuniformDIF}: \alpha_{J/k}^{(g)} = \alpha_{J/k} = \frac{\lambda_{J/k}^{(g)}}{\sigma_{J/k}} = \frac{\lambda_{J/k}}{\sigma_{J/k}}\\ & \text{for \textit{j} = 1,2,..,\textit{J}, \textit{c} = 0,1,2,...,\textit{C}-1 and for some \textit{k},}\\& \text{where \textit{k} = 1,2,...,\textit{J}}. \end{aligned} $$

Equation (16) shows the hypothesis of no nonuniform DIF indicating that for all items except item *k* the loadings are the same for all groups. Note that, without any further specification on identification constraints used to identify the baseline model, this test differs from testing metric invariance in MG-CCFA not only because items are evaluated individually but also due to the presence of both loadings *λ* and unique variances *σ*^2^. However, under the minimal identifiability constraints proposed by Chang et al., (2017), unique variances are constrained to be 1 and equal across groups, making this test equivalent to testing metric invariance in MG-CCFA but for each individual item.

### MI testing strategies

#### MG-CCFA-based

Besides commonly testing different hypotheses, MG-CCFA and MG-IRT differ in terms of what testing strategies/measures are used to test these hypotheses. Within MG-CCFA, the common strategy is to estimate two nested models and then compare how well they fit the data. A measure of how well a model fits the data is commonly obtained by means of a goodness-of-fit index, which measures the similarity between the model-implied covariance structure and the covariance structure of the data (Cheung and Rensvold, 2002). To date, many fit indices exist, and they can be mainly divided into three categories: measures of absolute fit, misfit, and comparative fit (for a more detailed review on the available measures we refer the reader to Schreiber et al.,, 2006).

##### Absolute fit indices

Absolute fit indices focus on the exact fit of the model to the data and one of the most commonly used is the Chi-squared (*χ*^2^) test. Imagine a MG-CCFA model A, with $\chi ^{2}_{ModA}$ and *df*_*ModA*_ indicating the model *χ*^2^ and *degrees of freedom*, which fits the data sufficiently well. To test one of the MI hypotheses (e.g., metric invariance), a new model is specified by constraining the parameters of interest (e.g., loadings) of all items to be equal across groups. Let us call this model B, with $\chi ^{2}_{ModB}$ and *df*_*ModB*_. A *χ*^2^ test is then conducted by looking at the difference in these two models:

17 $$ T \sim {\chi^{2}_{D}}(df_{D}) = \chi^{2}_{ModB}-\chi^{2}_{ModA}(df_{ModB}-df_{ModA}). $$

A significant T (e.g., using a significance level of .05) indicates that model B fits significantly worse, and thus that model A should be preferred. This implies that invariance of the constrained parameters (e.g., loadings) does not hold. Two considerable limitations of the *χ*^2^ test are that, on the one hand, its performance is largely underpowered for small samples because the test statistic is only *χ*^2^-distributed as *N* goes to infinity (i.e., only with large samples). On the other hand, it is highly strict with large samples, indicating, for example, that two models are significantly different even when the differences in the parameters are small.

##### Misfit indices

On top of the well-known limitations of the *χ*^2^ test, a general counterargument towards the use of absolute fit indices is that we might not be necessarily interested in the exact fit as much as the extent of misfit in the model (Millsap, 2012). In this case, misfit indices, such as the root mean square error approximation (RMSEA) can be used. This index quantifies the misfit per degrees of freedom in the model (Browne & Cudeck, 1993). Specifically, in the case of multiple groups, it can be expressed as:

18 $$ RMSEA = \sqrt{G}  \sqrt{ max \left[ \frac{\chi^{2}_{ModA}}{df_{ModA}}-\frac{1}{N-1}, 0 \right] }. $$

Based on which MI hypothesis is tested, different criteria and procedures are used to determine whether the RMSEA is acceptable. In the configural step, the absolute value of RMSEA is used. Specifically, values between 0 and .05 indicate a “good” fit, and values between .05 and .08 are thought to be a “fair” fit (Browne & Cudeck, 1993; Brown, 2014). In the subsequent steps, the change in the RMSEA (ΔRMSEA) between the constrained and the unconstrained model is used instead of the absolute value of the measure. Specifically, a ΔRMSEA of .01 has been suggested as a cut-off value in the case of metric invariance and, similarly, a value of .01 should be used for scalar invariance (Cheung & Rensvold, 2002; Chen, 2007). When the change in the ΔRMSEA is higher than the specific cut-off, invariance is rejected.

##### Comparative fit indices

The third category of fit indices is the one of comparative fit, where the improvement of the hypothesized model compared to the null model is used as an index to test MI. Differently from exact fit indices, where the hypothesized model is compared against a saturated model (a model with *df* = 0), in comparative fit indices a comparison is conducted between the hypothesized model and the null model, with $\chi ^{2}_{ModNull}$ and *df*_*ModNull*_. The latter is a model in which all the measured variables are uncorrelated (i.e., a model where there is no common factor). It is worth to note that numerous comparative fit measures exist and, among them, a well-known one is the comparative fit index (CFI) (Bentler, 1990). The CFI measures the overall improvement in the *χ*^2^ in the tested model compared to the null model, and can be formally written as:

19 $$ CFI = 1 - \frac{\chi^{2}_{ModA}- df_{ModA}}{\chi^{2}_{ModNull} - df_{ModNull}} $$

where a value of .95 is used as a cut-off value in the configural invariance step to indicate a “good” fit (Bentler, 1990). In the subsequent steps, the common guidelines for cut-off values focus on the change in CFI (ΔCFI). Specifically, a ΔCFI larger than -.01 is considered to be problematic both in the case of testing for loadings and thresholds invariance (Cheung & Rensvold, 2002; Chen, 2007). It is worth noting that the default baseline model used in most CFA software (e.g., *lavaan*; Rosseel, 2012) may not be appropriate for testing MI and different alternatives exist (Widaman & Thompson, 2003; Lai & Yoon, 2015). Moreover, it is not yet clear whether the commonly accepted cut-off values for CFI, or alternative fit measures, can be directly applied to models that are not estimated using maximum likelihood, and caution is thus recommended in empirical practice when making decisions based on various goodness-of-fit indices (Xia & Yang, 2019).

#### MG-IRT-based

In MG-IRT-based approaches both parametric and nonparametric methods exist to test for uniform and nonuniform DIF. In this paper, the focus is on parametric methods, where a statistical model is assumed. Specifically, methods that compare the models’ likelihood functions will be discussed (for a more detailed discussion on both parametric and nonparametric methods for DIF detection, we refer the reader to Millsap, 2012).

##### Likelihood-ratio test

One well-known technique for the study of DIF is the likelihood-ratio test (LRT) (Thissen et al., 1986; Thissen, 1988; Thissen et al., 1993). In this test, the log-likelihood of a model with the parameters of all items constrained to be equal across groups is compared against the log-likelihood of the same model with freed parameters for one item only. The former is sometimes called the compact model (*L*_*C*_), while the latter is sometimes called the augmented model (*L*_*A*_, Kim & Cohen, 1998; Finch, 2005). Once these two models are estimated and the log-likelihood (*lnL*_*C*_ and *lnL*_*A*_) is obtained, the test statistic (*G*^2^) can be calculate using the following formula:

20 $$ G^{2} = -2lnL_{C} - (-2lnL_{A}) = -2lnL_{C} + 2lnL_{A}. $$

Similarly to the Chi-squared test in MG-CCFA, the test statistic *G*^2^ is *χ*^2^ distributed with *df* equal to the difference in the number of parameters estimated in the two models (Thissen, 1988). The same procedure is then iteratively repeated for all items. It is important to highlight that the above equation represents an an omnibus test of DIF, which in case of a significant result could be further inspected by constraining only specific parameters. For example, it would be possible to test uniform DIF by allowing only the thresholds to vary across groups.

##### Logistic regression

Logistic regression (LoR; Swaminathan and Rogers, 1990) is another parametric approach that has recently gained interest among DIF experts (Yasemin et al., 2015). The intuition behind the LoR approach is similar to the one of step-wise regression in which one can test whether the model improves by sequentially entering new predictors. The common order in which the variables are introduced, starting with a null model where only the intercept is estimated, is by first adding the latent construct, then the grouping variable, and finally an interaction between the latent construct and the grouping variable. Formally, this sequence of models is written as:

21 $$ Model  0: logit P(y_{j} \geq c) = \nu_{c}; $$

22 $$ Model  1: logit P(y_{j} \geq c) = \nu_{c} + {\upbeta}_{1}\eta; $$

23 $$ Model  2: logit P(y_{j} \geq c) = \nu_{c} + {\upbeta}_{1}\eta + {\upbeta}_{2}G; $$

24 $$ Model  3: logit P(y_{j} \geq c) = \nu_{c} + {\upbeta}_{1}\eta + {\upbeta}_{2}G + {\upbeta}_{3}\eta G. $$

In the equations above, *P*(*y*_*j*_ ≥ *c*) is the probability of the score on item *j* falling in category *c* or higher, and *ν*_*c*_ is a category-specific intercept. It is worth pointing out that, compared to the LRT, the latent variable scores are in this case only estimated once and then treated as observed, which can be problematic. In fact, since the latent variable scores are estimated and not observed, there might be uncertainty in the estimates, which could, in turn, affect the performance of this method. Moreover, some alternative formulations make use of sum scores instead of estimates of latent variable scores (Rogers & Swaminathan, 1993). Once the logistic regression models are estimated and a *G*^2^ is obtained, an omnibus DIF test can be conducted by:

25 $$ G_{omnibus}^{2} = G_{Model3}^{2} - G_{Model1}^{2}, $$

which is asymptotically *χ*^2^ distributed with *df* = 2 (Swaminathan & Rogers, 1990). Zumbo (1999) suggested to investigate the source of bias by separately testing for uniform and nonuniform DIF, respectively:

26 $$ G_{uniDIF}^{2} = G_{Model2}^{2} - G_{Model1}^{2} $$

and:

27 $$ G_{nonuniDIF}^{2} = G_{Model3}^{2} - G_{Model2}^{2} $$

where both (26) and (27) are *χ*^2^ distributed with *df* = 1.

The omnibus test procedure (25) turned out to have an inflated number of incorrectly flagged DIF items (type I error; Li and Stout, 1996). To solve this issue, a combination of a significant 2-*df* LRT (25) and a measure of the magnitude of DIF using a pseudo-*R*^2^ statistic has been suggested as an alternative criterion (Zumbo, 1999). The underlying idea is to treat the β coefficients as weighted least squares estimates and look at the differences in pseudo-*R*^2^ (Δ*R*^2^) measures between the model with and without the added predictor (e.g., Cox & Snell 1989). Specifically, to flag an item as DIF, both a significant *χ*^2^ test (with *df* = 2) and an effect size measure with an Δ*R*^2^ of at least .13 is suggested to be used (Zumbo, 1999).

## Simulation studies

To evaluate the impact of MG-CCFA- and MG-IRT-based hypotheses and testing strategies on the power to detect violations of MI, two simulation studies were performed. In the first study, an invariance scenario was simulated where parameters were invariant between groups. In the second study, a non-invariance scenario was simulated where model parameters varied between groups.

### Simulation Study 1: invariance

In the first study, three main factors were manipulated:

1. The number of items at two levels: 5, 25, to simulate a short and a long scale;
2. The number of categories for each item at two levels: 3, 5;
3. The number of subjects within each group at two levels: 250, 1000.

These factors were chosen to represent situations that can be encountered in psychological measurement. For example, the two levels at which the scale length varies are representative of (i) short scales that are used as an initial screening or to save assessment time in case of multiple administrations (e.g., clinical setting), and (ii) long scales typically used to obtain a more detailed and clear evaluation of the measured psychological construct. For the number of categories, the two levels mimic items constructed to capture a less or more nuanced degree of an agreement. Finally, the two simulated sample sizes resemble studies with “relatively”small samples (e.g., clinical setting) and with large samples (e.g., cross-cultural research).

A full-factorial design was used with 2 (number of items) x 2 (number of categories) x 2 (number of subjects within each group) = 8 conditions. For each condition, 500 replications were generated.

#### Method

##### Data generation

Data were generated from a factor model with one factor and two groups. The population values of the model parameters were chosen prior to conducting the simulation study and are reported in Table 1. Note that, for both groups, the factor mean and variance was set to 0 and 1, respectively. The choice of the values began with specifying the standardized loadings. Specifically, they were selected to resemble the ones commonly found in real applications with items having medium to high correlation with the common factor but differing among them (Stark et al., 2006; Wirth & Edwards, 2007; Kim & Yoon, 2011).

**Table 1:** Population values used in the simulation study

Item				3 categories		5 categories			
	*λ*	*σ*^2^	*κ*	*τ*_1_	*τ*_2_	*τ*_1_	*τ*_2_	*τ*_3_	*τ*_4_
1	.5	0.75	0	-0.38	0.38	-0.84	-0.25	0.25	0.84
2	.7	0.51	0	0.12	0.88	-0.34	0.25	0.75	1.34
3	.6	0.64	0	-0.88	-0.12	-1.34	-0.75	-0.25	0.34
4	.4	0.84	0	-0.88	-0.12	-1.34	-0.75	-0.25	0.34
5	.3	0.91	0	0.12	0.88	-0.34	0.25	0.75	1.34

The second step was to select the thresholds and, in order to choose them, continuous data with 10,000 observations were firstly generated under a factor model using the loadings in Table 1. Afterwards, using the distribution of the item scores for item 1, which was subsequently used as the anchor item, the tertiles (for items with three categories) and the quintiles (for items with five categories) were calculated. Then, the generation of the remaining thresholds proceeded by shifting the tertiles/quintiles of the first item by half a standard deviation. In detail, for both the three- and five-categories case, we shifted the thresholds value of the second and fifth item by + .50 and of the third and fourth item by - .50 (as can be seen from Table 1). In the conditions with 25 items, the same parameters in Table 1 were repeated five times. For all estimated models, we used the minimal identification constraints described in Eqs. (7) through (10) to identify the baseline model, and item 1 was used as the anchor item.

#### Data analysis

##### Scale level

The specification of the MG-CCFA models to test MI followed the common steps of a general MI testing procedure as described in Sect. 2.2.1. Specifically, in the configural step, a unidimensional factor model was fitted to both groups allowing loadings and thresholds to differ between groups (configural invariant model). In the metric step, factor loadings were constrained to be equal across groups while allowing the thresholds to be freely estimated (metric invariant model). In the scalar step, both factor loadings and thresholds were constrained to be equal across groups (scalar invariant model). Afterwards, a *χ*^2^ test (*α* = .05) was conducted between: (i) the model estimated in the configural and the metric step to test for loadings invariance, and (ii) the model estimated in the metric and scalar step to test for thresholds invariance. Additionally, the change in RMSEA (ΔRMSEA) and in CFI (ΔCFI) was calculated between the just mentioned models. Loadings non-invariance was concluded if at least one of the following criteria were met: a significant *χ*^2^ test, a ΔRMSEA > .01 or a ΔCFI < -.01. Additionally, since the common guidelines reported in the literature recommend to base decisions about (non)invariance of parameters using various indices, a combined criterion was created. According to this combined criterion, loadings non-invariance at the scale level was concluded if both a significant *χ*^2^ test and at least one between a ΔRMSEA >. 01 or a ΔCFI < -.01 was found (Putnick & Bornstein, 2016). Thresholds non-invariance at the scale level was concluded if at least one of the following criteria was met: a significant *χ*^2^ test, a ΔRMSEA >. 01 or a ΔCFI < -.01. Also, in this case, a combined criterion was created. Specifically, a scale was considered non-invariant with respect to thresholds if both a significant *χ*^2^ and at least one between a ΔRMSEA >. 01 or a ΔCFI < -.01 was found. All MG-CCFA models were estimated using diagonally weighted least squares (DWLS), but the full weight matrix was used to compute the mean-and-variance-adjusted test statistics (default in *lavaan*; Rosseel, 2012). This is a two-step procedure, where in the first step the thresholds and polychoric correlation matrix are estimated, and then, in the second step, the remaining parameters are estimated using the polychoric correlation matrix from the previous step.

In MG-IRT-based procedures, MI is tested for each item individually. Therefore, to conduct a test at the scale level, we decided to flag the scale as non-invariant if at least one item was flagged as non-invariant, correcting for multiple testing. Two different testing strategies were considered: the logistic regression (LoR) procedure and the likelihood-ratio test (LRT). Within LoR, two different criteria were used to flag an item as non-invariant. The first criterion is based on the likelihood-ratio test (LRT). Specifically, an item was non-invariant, either with respect to loadings or thresholds, in the case of a significant *χ*^2^ test (*α* = .05) between a model where the latent construct score, the grouping variable and an interaction between the two are included (see formula 24) and a model with only the latent construct score (see formula 22) (Swaminathan & Rogers, 1990). The second criterion, which will from this point on be called *R*^2^, combines the just mentioned *χ*^2^ test with a measure of the magnitude of DIF. The latter is obtained by computing the difference between a pseudo-*R*^2^ measure between the two above-mentioned models (Δ*R*^2^). Using this approach, an item was flagged as non-invariant when both a significant *χ*^2^ test and a Δ*R*^2^ >.02 were found (Choi, Gibbons, & Crane, 2011). Specifically, in this simulation study, the McFadden pseudo-*R*^2^ measure was used (Menard, 2000). In the case of the LRT, two different models per item were estimated. In one model the constraints on the thresholds were released for a specific item (uniform DIF model), while in the other the constraint on the loading was released (nonuniform DIF model). Additionally, a model with all items constrained to be equal was estimated (fully constrained model). An item was flagged as non-invariant with respect to thresholds in case of a statistically significant 1-*df* LRT (*α* = .05) between the fully constrained model and the uniform DIF model. Similarly, an item was flagged as non-invariant with respect to loadings in case of a statistically significant 1-*df* LRT (*α* = .05) between the fully constrained model and the nonuniform DIF model. This procedure was repeated iteratively for all the other items. Since multiple tests are conducted for the scale, a Bonferroni correction was used.

##### Item level

In order to test MI at the item level using a MG-CCFA-based testing strategy, a backward/step-down procedure was used (Kim & Yoon, 2011; Brown, 2014). The rationale is the same as the one just described in the LRT for MG-IRT. Specifically, the constraints (either on the thresholds or on the loading) were released for only one item, while keeping all the other items constrained to be equal. Hence, for each item, two different models were estimated. Then, the *χ*^2^ test (*α* = .05) was conducted and the ΔRMSEA and ΔCFI calculated. This procedure was then repeated iteratively for all the other items. Note that, due to the multiple tests conducted, Bonferroni correction was used. For MG-IRT-based procedures, the same procedures and criteria used at the scale level were used to test MI at the item level (but without applying a Bonferroni correction).

##### Outcome measures

The convergence rate (CR) and the false-positive rate (FPR) were calculated both for MG-CCFA- and MG-IRT-based procedures both at the scale level and at the item level. The CR indicates the proportion of models that converged while the FPR represents the scales/items incorrectly flagged as non-invariant. If models did not converge, new data were generated and models were rerun in order to always calculate the FPR based on 500 repetitions.

##### Data simulation, software, and packages

The data were simulated and analyzed using R (Core Team, 2013). Specifically, for estimating MG-CCFA and obtaining fit measures, the R package *lavaan* was used (Rosseel, 2012), while for LoR and the LRT *lordif* (Choi et al., 2011) and *mirt* (Chalmers, 2012) were used, respectively.

#### Results

##### Convergence rate

The convergence rate was almost 100% for all the considered approaches across all the conditions. Models’ non-convergence was observed only for a few conditions with small sample size as well as short scales and never exceeded 1%. The tables showing the complete results can be found in the Appendix (Tables 10 through 13)

##### Scale-level performance

The scale-level results when loadings equivalence was tested are reported in Table 2. For MG-CCFA-based approaches, ΔRMSEA showed a FPR >.10 in the conditions with short scales, whereas, for ΔCFI, this discrepancy was observed only in the conditions with both small sample size and short scales. Within MG-IRT-based approaches, the results were quite different, depending on the testing strategy. For the LoR approach, using the LRT criterion, the results obtained in this simulation study align with the ones in the existing literature, with an evident inflation of the FPR (overall, FPR >.40) (Rogers & Swaminathan, 1993; Li & Stout, 1996). For the *R*^2^ criterion, where a combination of the LRT and a pseudo-*R*^2^ measure was used, the FPR was at or below the chosen *α* level using the *R*^2^ criterion, with an inflated FPR only in the case with *N* = 250, *C* = 3 and *J* = 5 (FPR = 0.182). One possible explanation is that, due to the small amount of information available for each person in this condition, there is more uncertainty in the estimated scores of the latent construct. Since these estimates are then used as observed variables in the LoR procedure, they are likely to produce a larger number of items incorrectly flagged as non-invariant. Finally, the LRT showed an acceptable FPR in all conditions when testing for loadings equivalence at the scale level.

**Table 2:** Loadings’ FPR scale level - invariance scenario

			FPR scale level - loadings						
			MG-CCFA				MG-IRT LoR		MG-IRT LRT
N	C	J	Comb	*χ*^2^	ΔRMSEA	ΔCFI	LRT	*R*^2^	LRT
250	**3**	**5**	0.052	0.052	0.165	0.167	0.577	0.182	0.030
		**25**	0.036	0.040	0.072	0.032	0.399	0.026	0.032
	**5**	**5**	0.034	0.034	0.194	0.178	0.502	0.022	0.026
		**25**	0.048	0.058	0.074	0.032	0.406	0	0.038
1000	**3**	**5**	0.046	0.048	0.100	0.024	0.628	0	0.032
		**25**	0.008	0.052	0.008	0	0.438	0	0.048
	**5**	**5**	0.042	0.046	0.102	0.020	0.546	0	0.038
		**25**	0.008	0.064	0.008	0	0.366	0	0.032

The bold entries were used to distinguish between design factors and do not refer to results

Note. MG-CCFA = Multiple-group categorical confirmatory factor analysis; MG-IRT LoR = Logistic regression with MG-IRT; MG-IRT LRT = Likelihood-ratio test with MG-IRT; *N* = Sample size within each group; C = Number of categories; J = Number of items; Comb = combination of *χ*^2^, ΔRMSEA and ΔCFI

The results of the simulation study when equivalence of thresholds was tested at the scale level are reported in Table 3. For MG-CCFA-based methods, the FPR was above .10 for ΔRMSEA in the conditions with short scales and for ΔCFI in the conditions with short scales and small sample size. The combined criterion and the *χ*^2^ test provided acceptable FPR rates across conditions. For MG-IRT-based testing strategies, the obtained results are similar to the ones observed in the case of testing loadings equivalence. Specifically, for the LoR approach, the *R*^2^ criterion performed well in all conditions except when *N* = 1000, *C* = 3 and *J* = 5 (FPR = .189). Moreover, the LRT criterion for LoR showed an evident inflation across all conditions. Finally, the LRT performed well in all conditions.

**Table 3:** Thresholds’ FPR scale level - invariance scenario

			FPR scale level - thresholds						
			MG-CCFA				MG-IRT LoR		MG-IRT LRT
N	C	J	Comb	*χ*^2^	ΔRMSEA	ΔCFI	LRT	*R*^2^	LRT
250	**3**	**5**	0.042	0.042	0.180	0.252	0.660	0.189	0.036
		**25**	0.020	0.042	0.014	0.014	0.404	0.020	0.032
	**5**	**5**	0.038	0.038	0.178	0.228	0.527	0.020	0.036
		**25**	0.036	0.050	0.048	0.020	0.370	0	0.042
1000	**3**	**5**	0.044	0.044	0.118	0.066	0.626	0.002	0.042
		**25**	0	0.046	0	0	0.442	0	0.030
	**5**	**5**	0.054	0.054	0.124	0.080	0.528	0	0.034
		**25**	0.002	0.040	0.002	0	0.384	0	0.036

The bold entries were used to distinguish between design factors and do not refer to results

Note. MG-CCFA = Multiple-group categorical confirmatory factor analysis; MG-IRT LoR = Logistic regression with MG-IRT; MG-IRT LRT = Likelihood-ratio test with MG-IRT; *N* = Sample size within each group; C = Number of categories; J = Number of items; Comb = combination of *χ*^2^, ΔRMSEA and ΔCFI

##### Item-level performance

The results when loadings equivalence was tested at the item level are reported in Table 4. For MG-CCFA, all fit measures performed well as indicated by the FPRs that were close to the nominal *α* level. For MG-IRT using the LoR procedure, the LRT criterion produced a high number of false positives with short scales. Moreover, the results for both the *R*^2^ criterion and the LRT were within the chosen *α* level in almost all conditions, and never exceeded 0.06.

**Table 4:** Loadings’ FPR item level - invariance scenario

			FPR item level - loadings						
			MG-CCFA				MG-IRT LoR		MG-IRT LRT
N	C	J	Comb	*χ*^2^	ΔRMSEA	ΔCFI	LRT	*R*^2^	LRT
250	**3**	**5**	0.039	0.046	0.077	0.060	0.243	0.053	0.047
		**25**	0.002	0.055	0.002	0	0.022	0.001	0.050
	**5**	**5**	0.050	0.061	0.089	0.058	0.202	0.005	0.051
		**25**	0.002	0.059	0.002	0	0.020	0	0.049
1000	**3**	**5**	0.025	0.047	0.031	0.006	0.239	0	0.045
		**25**	0	0.052	0	0	0.021	0	0.057
	**5**	**5**	0.028	0.058	0.038	0.002	0.200	0	0.059
		**25**	0	0.052	0	0	0.021	0	0.047

The bold entries were used to distinguish between design factors and do not refer to results

Note. MG-CCFA = Multiple-group categorical confirmatory factor analysis; MG-IRT LoR = Logistic regression with MG-IRT; MG-IRT LRT = Likelihood-ratio test with MG-IRT; *N* = Sample size within each group; C = Number of categories; J = Number of items; Comb = combination of *χ*^2^, ΔRMSEA and ΔCFI

Finally, the results when testing thresholds equivalence at the item level are reported in Table 5. For MG-CCFA, all criteria performed reasonably well with some small inflations for ΔCFI in the conditions with small sample size and short scales. For MG-IRT-based testing strategies, only the LRT criterion for the LoR approach showed a FPR higher than the chosen *α* level with *J* = 5.

**Table 5:** Thresholds’ FPR item level - invariance scenario

			FPR item level - thresholds						
			MG-CCFA				MG-IRT LoR		MG-IRT LRT
N	C	J	Comb	*χ*^2^	ΔRMSEA	ΔCFI	LRT	*R*^2^	LRT
250	**3**	**5**	0.048	0.056	0.072	0.100	0.236	0.053	0.051
		**25**	0	0.048	0	0	0.022	0.001	0.053
	**5**	**5**	0.046	0.050	0.080	0.108	0.194	0.010	0.048
		**25**	0	0.050	0	0	0.020	0	0.050
1000	**3**	**5**	0.028	0.052	0.032	0.015	0.256	0	0.048
		**25**	0	0.051	0	0	0.021	0	0.049
	**5**	**5**	0.034	0.052	0.032	0.017	0.179	0	0.040
		**25**	0	0.049	0	0	0.020	0	0.048

The bold entries were used to distinguish between design factors and do not refer to results

Note. MG-CCFA = Multiple-group categorical confirmatory factor analysis; MG-IRT LoR = Logistic regression with MG-IRT; MG-IRT LRT = Likelihood-ratio test with MG-IRT; *N* = Sample size within each group; C = Number of categories; J = Number of items; Comb = combination of *χ*^2^, ΔRMSEA and ΔCFI

### Simulation Study 2: Non-invariance

In the second simulation study, three more factors were included to evaluate the performance of the studied approaches, with their respective testing strategies, in detecting violations of MI when parameters were non-invariant across groups. On top of varying the scale length, the number of categories and the sample size we now also vary:

1. Percentage of items with non-invariant loadings at 3 levels: 20%, 40% aligned, and 40% misaligned;
2. Percentage of items with non-invariant thresholds at 3 levels: 20%, 40% aligned, and 40% misaligned;
3. The amount of bias imposed for each non-invariant parameter at two levels: small and large.

The first three factors (i.e., number of items, number of categories for each item and number of subjects within each group) were the ones used in the previous simulation study. Additionally, to simulate differences in loadings/thresholds across groups, the values of the parameters were changed either for 20% or 40% of the items. Moreover, in the condition with 40% of the items having non-invariant loadings, the values were either increased for all items (e.g., all loadings on one group are higher), or increased for half of the items and decreased for the other half (e.g., in the condition with five items, where the values of two loadings are changed, one was increased and the other decreased). The former was labeled as an aligned change while the latter as a misaligned change.

The same procedure was followed for the shifts in thresholds both in terms of percentage of items with non-invariant thresholds and for the aligned or misaligned shifts. Note that, since each item has more than one threshold, all the thresholds of that item were shifted.

The percentages of items showing non-invariant loadings/thresholds were chosen to represent situations that can be observed in psychological measurement. For instance, situations with a well-functioning scale where only one item (in the case of short scales) or a few items (in the case of long scales) seem to function differently across groups or, alternatively, situations with a bad functioning scale where almost half of the items function differently across groups. Aligned differences were simulated to represent scales where items favor only one group, while misaligned differences mimic a situation where different items favor different groups.

The manipulated violations of MI, both for loadings and thresholds, were either small or large in order to represent both semi-bad functioning items and bad functioning items. On the one hand, a difference of .1 or .2 was used to simulate small and large changes in the standardized factor loadings, respectively. The chosen values substantially increase the variance accounted by the factor for the item. For example, in a standardized factor loading of .7 the explained variance of the item by the factor is .7^2^ = .49. If the loading is increased by .1 the explained variance will then be .8^2^ = .64. Also, in case of a big change (.2), the explained variance will become .9^2^ = .81. On the other hand, for the shifts in thresholds, the parameters of one group were shifted by either a quarter (.25) or half a standard deviation (.50) to simulate small and large violations of thresholds non-invariance.

In total, 2 (number of items) x 2 (number of categories) x 2 (number of subjects within each group) x 3 (percentage of non-invariant loadings) x 3 (percentage of non-invariant thresholds) x 2 (amount of bias imposed) = 144 conditions were simulated for the conditions with non-invariance in the loadings and the thresholds. For each condition, 500 replications were generated.

### Method

#### Data analysis

Like in the first simulation study, the data were generated from a factor model with one factor and two groups. The population parameters were the same as used in the first simulation study and they were varied, based on the condition, as just explained above. Moreover, the procedures used to specify and estimate the models, both at the scale and at the item level, were the same ones used previously. Differently from before, only a subset of the criteria was used to flag a scale/item as non-invariant. Specifically, only the criteria that showed an acceptable FPR across all conditions in the first simulation study are reported. This was done because procedures with unacceptable FPRs should not be considered for testing MI, and hence considering them here would not make sense. Thus, for MG-CCFA, only the results of the combined criterion and *χ*^2^ test are reported, while for MG-IRT-based procedures the LRT approach and, for the LoR approach, only the results of the *R*^2^ criterion.

#### Outcome measures

The convergence rate (CR), true-positive rate (TPR) and false-positive rate (FPR) were calculated both for the MG-CCFA- and MG-IRT-based procedures both at the scale and at the item level. Here, the TPR represents the proportion of non-invariant scales/items that are correctly identified as such, while the FPR represents the proportion of non-invariant scales/items that are incorrectly identified as such. If models did not converge, new data were generated and models were rerun in order to always calculate the TPR and the FPR for 500 repetitions.

### Results

**Convergence rate**

#### Scale level

The results of the CR when testing loadings equivalence at the scale level in the non-invariance scenario are displayed in Table 14 in the Appendix. In the conditions with large sample size, the CR when testing loadings equivalence at the scale level was above 99% for all the approaches. Compared to the conditions with a large sample size, the CR dropped in the conditions with small sample size and 40% of the items showing large misaligned changes in loadings. Specifically, the CR for MG-CCFA was .978 when *J* = 5 and *C* = 3 while for MG-IRT using the LoR approach the CR was around .9 with *N* = 250, *J* = 25 and both for items that had 3 or 5 categories.

The results of the CR when testing thresholds equivalence at the scale level in the non-invariance scenario are displayed in Table 15 in the Appendix. For MG-CCFA, the CR was generally lower in the conditions with large shifts in the thresholds compared to the conditions with small shifts. For example, with *N* = 250, *C* = 3, *J* = 5, and large misaligned shifts in the thresholds parameters the CR was .828. This lower CR could be due to a specific issue with the estimation procedure. In fact, using DWLS, the estimation heavily relies on the first step, where the thresholds and the polychoric correlation matrix are estimated. Large differences in thresholds between the two groups might affect this first step and, in turn, the remaining part of the procedure. On the contrary, for MG-IRT-based approaches, the CR was always above 99%.

#### Item level

The results of the CR when testing loadings equivalence at the item level in the non-invariance scenario are displayed in Table 16 in the Appendix. These results closely resemble the ones observed when loading equivalence was tested at the scale level. Specifically, the CR was below .98 for MG-CCFA only in the condition with *N* = 250, *C* = 3, *J* = 5, and large misaligned changes in loadings in 40% of the items. Moreover, for MG-IRT using the LoR approach the CR was around .89 when *N* = 250, *J* = 25, and with large misaligned changes in the loadings, regardless of the number of categories for each item.

The results of the CR when testing thresholds equivalence at the item level in the non-invariance scenario are displayed in Table 17 in the Appendix. For MG-CCFA, similar to what was observed at the scale level, the CR dropped in the conditions with small sample size, big shifts in thresholds and short scales compared to the other conditions. For example, the lowest CR was observed in the condition with *N* = 250, *C* = 3, *J* = 5 and large misaligned shifts in thresholds (CR = 0.798). However, for MG-IRT-based approaches the CR was always above 99%.

#### Scale-level performance

The results of the TPR when testing loadings equivalence at the scale level in the non-invariance scenario are displayed in Table 6. Although none of the approaches was particularly sensitive to small changes in loadings, the *χ*^2^ test often outperformed the other testing strategies in all conditions. For MG-CCFA, in addition to the *χ*^2^ test, a combined criterion was used to flag scales or items as non-invariant, and Table 20 in the Appendix displays the TPRs for each of the measures that form this combined criterion. For ΔCFI, the results seemed to highly depend on the length of a scale. In fact, for long scales, when small loading differences were simulated and the sample size was large, the TPRs drastically dropped reaching values generally close to 0. Also, since in the first simulation study the LoR approach with *N* = 250, *J* = 5 and *C* = 3 had an unacceptable FPR, the results in this simulation study are reported in red indicating that they should not be considered.

**Table 6:** Loadings’ TPR scale level - non-invariance scenario

				TPR scale level - loadings							
				MG-CCFA				MG-IRT			
				Comb		*χ*^2^		LoR		LRT	
N	C	J	%	small	large	small	large	small	large	small	large
250	**3**	**5**	**20%**	0.052	0.044	0.052	0.044			0.048	0.043
			**40%**	0.078	0.124	0.078	0.124			0.054	0.079
			**40% ±**	0.082	0.218	0.082	0.218			0.048	0.088
		**25**	**20%**	0.124	0.284	0.140	0.332	0.030	0.092	0.076	0.094
			**40%**	0.118	0.474	0.144	0.532	0.044	0.176	0.064	0.166
			**40% ±**	0.272	0.916	0.306	0.922	0.075	0.365	0.109	0.300
	**5**	**5**	**20%**	0.054	0.048	0.054	0.048	0.018	0.018	0.048	0.030
			**40%**	0.076	0.122	0.076	0.122	0.032	0.052	0.054	0.086
			**40% ±**	0.124	0.268	0.124	0.268	0.052	0.103	0.080	0.154
		**25**	**20%**	0.126	0.410	0.164	0.474	0	0.008	0.062	0.164
			**40%**	0.182	0.692	0.218	0.764	0.002	0.020	0.080	0.256
			**40% ±**	0.274	0.972	0.358	0.986	0.002	0.118	0.114	0.376
1000	**3**	**5**	**20%**	0.060	0.084	0.062	0.094	0	0	0.044	0.098
			**40%**	0.130	0.366	0.140	0.384	0	0.032	0.084	0.322
			**40% ±**	0.204	0.714	0.206	0.714	0.004	0.064	0.092	0.506
		**25**	**20%**	0.136	0.712	0.390	0.974	0	0	0.138	0.584
			**40%**	0.256	0.940	0.622	1	0	0	0.216	0.718
			**40% ±**	0.500	1	0.892	1	0	0.008	0.298	0.980
	**5**	**5**	**20%**	0.054	0.106	0.060	0.110	0	0	0.052	0.128
			**40%**	0.164	0.500	0.182	0.542	0	0	0.108	0.440
			**40% ±**	0.238	0.852	0.262	0.860	0	0.006	0.144	0.692
		**25**	**20%**	0.174	0.872	0.478	0.998	0	0	0.186	0.720
			**40%**	0.342	0.990	0.732	1	0	0	0.260	0.858
			**40% ±**	0.758	1	0.976	1	0	0	0.398	1

The bold entries were used to distinguish between design factors and do not refer to results

Note. MG-CCFA = Multiple-group categorical confirmatory factor analysis; MG-IRT LoR = Logistic regression with MG-IRT; MG-IRT LRT = Likelihood-ratio test with MG-IRT; *N* = Sample size within each group; C = Number of categories; J = Number of items; % = percentage of items affected by DIF (± misaligned); small = small bias; large = large bias; values in red = FPR ≥.10 in the invariance scenario

The results of the TPR when testing thresholds equivalence at the scale level in the non-invariance scenario are displayed in Table 7, and the results for each of the fit measures forming the combined criterion are displayed in the Appendix in Table 21. The *χ*^2^ test for MG-CCFA was remarkably sensitive to differences in thresholds and outperformed all the other approaches, regardless of other simulated conditions. In addition, LoR’s TPR was lower than the one of MG-CCFA and the LRT, in almost all conditions, and especially when the sample size was large. However, in the case of large misaligned shifts, the TPR was almost always the same as it was for MG-CCFA and the LRT.

**Table 7:** Thresholds’ TPR scale level - non-invariance scenario

				TPR scale level - thresholds							
				MG-CCFA				MG-IRT			
				Comb		*χ*^2^		LoR		LRT	
N	C	J	%	small	large	small	large	small	large	small	large
250	**3**	**5**	**20%**	0.358	0.908	0.358	0.908			0.131	0.448
			**40%**	0.720	1	0.720	1			0.285	0.759
			**40% ±**	0.652	0.996	0.654	0.996			0.246	0.864
		**25**	**20%**	0.414	1	0.742	1	0.144	0.932	0.264	0.884
			**40%**	0.392	1	0.716	1	0.168	0.948	0.268	0.902
			**40% ±**	0.906	1	0.996	1	0.832	1	0.468	0.996
	**5**	**5**	**20%**	0.396	0.974	0.396	0.974	0.076	0.449	0.104	0.512
			**40%**	0.766	1	0.766	1	0.118	0.475	0.230	0.800
			**40% ±**	0.806	1	0.806	1	0.319	0.989	0.271	0.911
		**25**	**20%**	0.560	1	0.738	1	0.022	0.602	0.254	0.922
			**40%**	0.630	1	0.742	1	0.032	0.592	0.244	0.876
			**40% ±**	0.996	1	1	1	0.612	1	0.400	0.996
1000	**3**	**5**	**20%**	0.956	1	0.956	1	0.026	0.474	0.550	1
			**40%**	1	1	1	1	0.022	0.571	0.888	1
			**40% ±**	1	1	1	1	0.202	1	0.978	1
		**25**	**20%**	0.828	1	1	1	0	0.556	0.954	1
			**40%**	0.802	1	1	1	0	0.556	0.944	1
			**40% ±**	1	1	1	1	0.626	1	1	1
	**5**	**5**	**20%**	0.984	1	0.984	1	0	0.226	0.598	1
			**40%**	1	1	1	1	0	0.220	0.910	1
			**40% ±**	1	1	1	1	0.018	1	0.986	1
		**25**	**20%**	0.980	1	1	1	0	0.024	0.958	1
			**40%**	0.972	1	1	1	0	0.030	0.964	1
			**40% ±**	1	1	1	1	0.430	1	1	1

The bold entries were used to distinguish between design factors and do not refer to results

Note. MG-CCFA = Multiple-group categorical confirmatory factor analysis; MG-IRT LoR = Logistic regression with MG-IRT; MG-IRT LRT = Likelihood-ratio test with MG-IRT; *N* = Sample size within each group; C = Number of categories; J = Number of items; % = percentage of items affected by DIF (± misaligned); small = small bias; large = large bias; values in red = FPR ≥.10 in the invariance scenario

#### Item-level performance

The results of the TPR when testing loadings equivalence at the item level in the non-invariance scenario are displayed in Table 8. The results of the FPR were also calculated and are displayed in Table 18 in the Appendix. The *χ*^2^ test often resulted in a TPR higher than the other approaches in all conditions. However, for this test, the FPR was generally >.1, especially in conditions with large sample size; we marked these TPRs with *, to indicate that these results should be interpreted with caution. Similar to the scale-level results, all testing strategies hardly detect non-invariance when small changes in the loadings were simulated for short scales, reaching a maximum TPR of .267 in the condition with misaligned changes affecting 40% of the items, *N* = 1000 and *C* = 5. Difficulties in flagging non-invariant items were even more pronounced in the conditions with long scales for the combined criterion, showing that loadings nonequivalence was not detected in most cases. The performance of each of the fit measures forming this criterion, for MG-CCFA, was further investigated. These results are displayed in the appendix in Table 22. For both ΔRMSEA and ΔCFI, when small loading changes were simulated, the results seemed to highly depend on the length of a scale. In fact, for long scales, both measures rarely detected changes in loadings. For MG-IRT-based approaches, differences in loadings were rarely detected by the LoR approach regardless of the condition, and with even lower frequencies when the sample size increases. The LRT outperformed LoR in all conditions in terms of the TPR.

**Table 8:** Loadings’ TPR item level - non-invariance scenario

				TPR item level - loadings							
				MG-CCFA				MG-IRT			
				Comb		*χ*^2^		LoR		LRT	
N	C	J	%	small	large	small	large	small	large	small	large
250	**3**	**5**	**20%**	0.038	0.060	0.052	0.076	0.004	0.004	0.061	0.064
			**40%**	0.052	0.088	0.061	0.103	0.055	0.088	0.067	0.116
			**40% ±**	0.077	0.192	0.091	0.205	0.063	0.119	0.068	0.142
		**25**	**20%**	0.007	0.015	0.107	0.259	0.004	0.019	0.087	0.224
			**40%**	0.003	0.007	0.078	0.162*	0.003	0.015	0.084	0.200
			**40% ±**	0.006	0.037	0.147	0.426	0.006	0.041	0.096	0.252
	**5**	**5**	**20%**	0.066	0.084	0.080	0.106	0	0	0.074	0.114
			**40%**	0.054	0.130	0.060	0.135	0.005	0.028	0.071	0.173
			**40% ±**	0.095	0.277	0.111	0.291	0.016	0.056	0.085	0.205
		**25**	**20%**	0.005	0.016	0.129	0.317	0	0.002	0.110	0.251
			**40%**	0.005	0.003	0.094	0.194*	0	0.002	0.111	0.230
			**40% ±**	0.008	0.032	0.172	0.533	0.001	0.012	0.111	0.303
1000	**3**	**5**	**20%**	0.042	0.074	0.096	0.182	0	0	0.098	0.178
			**40%**	0.071	0.213	0.114	0.318*	0.001	0.013	0.109	0.338
			**40% ±**	0.155	0.486	0.224	0.645*	0.001	0.045	0.136	0.421
		**25**	**20%**	0	0.001	0.274	0.705*	0	0	0.250	0.618
			**40%**	0	0	0.160*	0.465*	0	0	0.217	0.621
			**40% ±**	0	0.003	0.422	0.932	0	0.001	0.261	0.707
	**5**	**5**	**20%**	0.042	0.134	0.114	0.238	0	0	0.112	0.206
			**40%**	0.092	0.256	0.146	0.382*	0	0	0.156	0.454
			**40% ±**	0.174	0.526	0.267	0.754*	0	0.002	0.159	0.507
		**25**	**20%**	0.001	0	0.323	0.818*	0	0	0.283	0.725
			**40%**	0	0	0.207*	0.559*	0	0	0.288	0.732
			**40% ±**	0	0.003	0.491	0.978	0	0	0.298	0.812

The bold entries were used to distinguish between design factors and do not refer to results

Note. MG-CCFA = Multiple-group categorical confirmatory factor analysis; MG-IRT LoR = Logistic regression with MG-IRT; MG-IRT LRT = Likelihood-ratio test with MG-IRT; *N* = Sample size within each group; C = Number of categories; J = Number of items; % = percentage of items affected by DIF (± misaligned); small = small bias; large = large bias; * = FPR ≥.10

The results of the TPR when testing thresholds equivalence at the item level in the non-invariance scenario are displayed in Table 9. The results of the FPR were also calculated and are displayed in Table 19 in the Appendix. The *χ*^2^ test for MG-CCFA generally outperformed all the remaining approaches, regardless of the other factors. In addition, small differences in thresholds in the conditions with *N* = 1000 were rarely (or never) detected by the MG-CCFA-based combined criterion. Again, we inspected the TPR for each of the MG-CCFA-based fit measures that formed this criterion, and the results are displayed in Table 23 in the Appendix. The ΔRMSEA and ΔCFI TPRs were heavily affected by the length of the scale, and both criteria rarely flagged non-invariant items, especially in the conditions where small threshold differences were simulated.

**Table 9:** Thresholds’ TPR item level - non-invariance scenario

				TPR item level - thresholds							
				MG-CCFA				MG-IRT			
				Comb		*χ*^2^		LoR		LRT	
N	C	J	%	small	large	small	large	small	large	small	large
250	**3**	**5**	**20%**	0.536	0.976	0.586	0.988	0.014	0.112	0.214	0.660
			**40%**	0.643	0.986	0.647	0.986	0.059	0.289	0.312	0.763
			**40% ±**	0.555	0.984	0.566	0.984	0.239	0.543	0.293	0.784
		**25**	**20%**	0.003	0.143	0.648	0.997	0.010	0.372	0.349	0.886
			**40%**	0.002	0.127	0.646	0.997	0.019	0.342	0.336	0.886
			**40% ±**	0	0.130	0.657	0.998	0.153	0.655	0.360	0.885
	**5**	**5**	**20%**	0.626	0.994	0.674	0.996	0.002	0.011	0.198	0.738
			**40%**	0.689	0.999	0.696	0.999	0.018	0.084	0.305	0.810
			**40% ±**	0.675	0.999	0.678	0.999	0.131	0.503	0.309	0.813
		**25**	**20%**	0.006	0.368	0.724	0.999	0.002	0.098	0.362	0.880
			**40%**	0.008	0.360	0.724	0.999	0.001	0.098	0.353	0.879
			**40% ±**	0.004	0.357	0.726	0.998	0.100	0.526	0.339	0.875
1000	**3**	**5**	**20%**	0.978	1	0.988	1	0	0	0.758	1
			**40%**	0.993	1	0.999	1	0	0.055	0.869	1
			**40% ±**	0.976	1	0.994	1	0.116	0.500	0.857	0.998
		**25**	**20%**	0	0.117	1	1	0	0.157	0.918	1
			**40%**	0	0.146	0.997	1	0	0.182	0.908	1
			**40% ±**	0	0.124	0.998	1	0.072	0.579	0.920	1
	**5**	**5**	**20%**	0.998	1	0.998	1	0	0	0.808	1
			**40%**	0.998	1	1	1	0	0	0.894	1
			**40% ±**	0.990	1	0.998	1*	0.009	0.500	0.889	1
		**25**	**20%**	0	0.648	0.999	1	0	0.004	0.904	1
			**40%**	0	0.664	1	1	0	0.007	0.903	1
			**40% ±**	0	0.620	1	1	0.046	0.497	0.907	1

The bold entries were used to distinguish between design factors and do not refer to results

Note. MG-CCFA = Multiple-group categorical confirmatory factor analysis; MG-IRT LoR = Logistic regression with MG-IRT; MG-IRT LRT = Likelihood-ratio test with MG-IRT; *N* = Sample size within each group; C = Number of categories; J = Number of items; % = percentage of items affected by DIF (± misaligned); small = small bias; large = large bias; * = FPR ≥.10

### Conclusions

Based on the results observed in the invariance scenario, we can conclude that, for only some of the MG-CCFA- and MG-IRT-based testing strategies a FPR below or at the chosen *α* level was found. In fact, among the considered testing strategies used to flag a scale/item as non-invariant, quite many methods had an inflated type I error. For MG-CCFA-based criteria, the FPR was often below or at the chosen *α* level for the *χ*^2^ test or when a combination of a *χ*^2^ test and an alternative fit measure (e.g., RMSEA or CFI) was used. For MG-IRT-based approaches, the LRT provided a well-controlled FPR in all conditions regardless of whether the test was conducted at scale or at the item level. The LoR approach for MG-IRT showed an inflated FPR when the LRT criterion was used, while adopting a combination of both the LRT criterion and a pseudo-*R*^2^ measure resulted in a low FPR in (almost) all conditions.

Based on the results observed in the non-invariance scenario, we can conclude that, when testing loadings equivalence, small changes in loadings are hard to detect regardless of whether a test is performed at the scale level or at the item level. Furthermore, the *χ*^2^ test generally outperformed MG-IRT-based testing strategies when loadings non-invariance was tested at the scale level, whereas the LRT outperformed MG-CCFA-based testing strategies and LoR when loadings non-invariance was tested at the item level. In fact, while the item-level *χ*^2^ test was more sensitive than the item-level LRT to changes in loadings, the FPR for the *χ*^2^ test was generally above the nominal *α* level, and especially high in conditions with large sample size. The latter result is in line with previous literature, which suggested that the item-level LRT outperforms MG-CCFA-based approaches when considering both TPR and FPR (Kim & Yoon, 2011). Therefore, in empirical practice, the item-level LRT might be preferred if one aims at testing loading equivalence for each item separately. In addition, when testing threshold equivalence, the *χ*^2^ test outperformed all the other testing strategies both when MI was tested at the scale and item level. Furthermore, in the non-invariance scenario, for MG-CCFA, a combined criterion was used to flag scales/items as non-invariant, and we further inspected the TPRs for each of the measures that form this combined criterion. These results, for the scale- and item-level tests, are displayed in the Appendix in Tables 20 through 23, respectively. In particular, the TPRs for ΔRMSEA and ΔCFI were heavily affected by both scale length and the level at which MI was tested (scale or item). Specifically, for long scales, these two measures hardly detected changes in loadings and thresholds, especially when the test was conducted at the item level.^1^ This result is especially relevant in empirical practice, where researchers commonly base MI decisions on multiple criteria (Putnick & Bornstein, 2016). Based on our results, we would discourage researchers to use any of these fit measures, in particular when testing MI for each item individually.

## Discussion

When comparing psychological constructs across groups, testing for measurement invariance (MI) plays a crucial role. With ordinal data, multiple group categorical confirmatory factor analysis (MG-CCFA) and multiple group item response theory (MG-IRT) models can be made equivalent using a set of minimal identification constraints (Chang et al., 2017). Still, differences between these two approaches exist in the context of MI testing. These differences are reflected in: (i) the hypotheses being tested, and (ii) the testing strategies/measures used to test these hypotheses. In this paper, two simulation studies were conducted to evaluate the performance of the different testing strategies and measures in testing MI when: (i) the test is conducted at the scale or at the item level and, (ii) MG-CCFA- or MG-IRT-based testing strategies are used. In the first simulation study, an invariance scenario was simulated where no differences existed in the parameters across groups. In addition, a second simulation study was conducted to assess the performance of these approaches when non-invariance was simulated between groups.

A key result of these simulation studies is that MG-CCFA-based testing strategies are generally better than MG-IRT-based ones when testing for MI at the scale level. Therefore, in empirical practice, we recommend using either the *χ*^2^ test or a combination of a *χ*^2^ test with an alternative fit measure (i.e., RMSEA or CFI) when testing MI at the scale level. In addition, when testing MI at the item level, the *χ*^2^ test performed better than MG-IRT-based approaches when threshold equivalence was tested, whereas, when loading equivalence was tested, the item-level LRT provided the best trade-off between correctly and incorrectly identified non-invariant items.

In addition, another key result pertains to how the length of a scale and the level at which MI is tested affects the performance of MG-CCFA’s fit measures. In fact, both RMSEA and CFI hardly detected non-invariant parameters when MI was tested for each item individually, especially with long scales. That is, the more items on a scale, the harder it is for these measures to detect whether a specific item is non-invariant. These results identify a fundamental issue when using these fit measures to test MI at the item level. In fact, the cut-off values that are commonly used seem to be inadequate for item-level testing, since their performance heavily depends on the scale’s length. Commonly, MG-CCFA is used to test for MI at the scale level, which might explain why most papers focused on defining optimal cut-off values for these measures when MI is tested at this level (Cheung & Rensvold, 2002; Chen, 2007; Rutkowski & Svetina, 2014; 2017). If non-invariance is detected, researchers might decide to inspect its source by conducting a test for each item individually (Kim & Yoon, 2011; Putnick & Bornstein, 2016). Based on our results, we would discourage researchers from using such measures to this aim since the cut-off values need to be re-evaluated for item-level testing in future research. In this sense, dynamic procedures for determining fit-indices cut-off values, where appropriate cut-off value are derived based on a specific model (McNeish & Wolf, 2020), are a promising solution, and it is especially important to extend and evaluate these procedures to MI testing with ordered-categorical data. Finally, to obtain indications on whether and where DIF exist, modification indices might help; however, the performance of such tools in determining non-invariant items remains unclear and requires further research.

The simulation studies conducted provide a useful indication in terms of the performance of testing strategies and measures in testing MI for models applied to ordinal data. Still, they are not free of limitations, and it is relevant to highlight some of those. An important limitation of our work has to do with the assumptions that are made by the different measurement models. While the imposed constraints and testing steps we followed can be considered standard, using these constraints may prevent a more fine-grained analysis of MI. Specifically, to validly compare MG-CCFA- and MG-IRT-based approaches, it was crucial that MI was tested using an equivalent measurement model, which was specified using the set of constraints proposed by Chang et al., (2017). These constraints can be seen as MG-GRM-type constraints, where both the unique variances and the intercepts are constrained to be equal across groups. Imposing such equalities, which is commonly done in MG-IRT-based approaches, could be limiting if the goal is to have a more fine-grained analysis of MI. Furthermore, MG-CCFA-based constraints may be better suited to distinctly unravel differences in unique variances and intercepts across groups, and Wu and Estabrook (2016) have recently shown that, within the MG-CCFA framework, it may be preferable to select identification constraints based on which parameters are tested for non-invariance in order to avoid model misspecification. In detail, the authors showed that, for MG-CCFA, constraints that are commonly imposed on a baseline model (i.e., the configural model, where equal number of factors and loadings structure are imposed across groups) can become restrictions when new invariance constraints (e.g., constraining all loadings to be equal) are added. As a consequence, it may be preferable to define a baseline model on a case-by-case basis depending on the type of invariance tested (e.g., thresholds invariance). Therefore, we strongly recommend researchers to carefully evaluate the suitability of the restrictions underlying classical MG-CCFA- and MG-IRT-based procedures such as the ones presented here before testing for MI.

Another important set of limitations pertains to the dimensionality of the simulated scales as well as the lack of unique covariances. In particular, we focused on unidimensional scales, while researchers are frequently confronted with scales that capture multiple dimensions. Generally, MG-CCFA is used for multidimensional constructs, while MG-IRT-based models are preferred with unidimensional constructs. It might therefore be interesting to inspect if similar results as the ones observed here would be obtained when model complexity is increased by having multiple dimensions. In addition, the data-generating models did not include any residual covariances among items, which are not uncommon in empirical practice (MacCallum & Tucker, 1991). Ignoring such residual covariances by assuming uncorrelated errors can affect MI testing for continuous data (Joo & Kim, 2019) but further research should focus on assessing how residual covariances affect MI testing for ordered-categorical data.

Another set of limitations pertains to the grouping. Firstly, in the current simulation studies, we inspected the performance of MG-CCFA- and MG-IRT-based testing strategies with only two groups. However, cross-cultural and cross-national data, where many groups are compared simultaneously, are rapidly increasing in psychological sciences. For this reason, it might be useful to investigate differences in the performance of the studied approaches when many groups are compared. Secondly, in these simulation studies, we knew which subject belonged to which group, and differences were created between the groups’ measurement models. However, the grouping of subjects is not always known and/or researchers might not have access to those variables that are thought to cause heterogeneity (e.g., nationality, gender). In this case, a different approach might be preferred to disentangle the heterogeneity across participants (e.g., factor mixture models; Lubke, 2005).

One last important set of limitations concerns the anchoring of the scale. That is, which items’ parameters are set equal across groups in order to identify the model and to make the scale comparable across groups. First, the item that was used as the anchor in the simulation studies was known to be invariant across groups. In real applications, this information is never known beforehand, and estimating a model relying on an inadequate anchor item could impact model’s convergence as well as the ability to detect non-invariance of parameters. This issue has been partly discussed in previous studies comparing different type of identification constraints (Chang et al., 2017). It could be interesting to inspect how the choice of a “good” or “bad” anchor item influences the detection of MI in a more comprehensive study. Second, in these simulation studies, a set of minimal constraints was used to make the measurement models equivalent, and only one item was constrained to be equal across groups. Minimal constraints allow most parameters to be freely estimated. However, when specific items are known to function similarly across groups (e.g., knowledge based on prior studies or strong motivations to consider them invariant across groups) it might be beneficial, both in terms of the estimation and the power to detect non-invariance of the model’s parameters, to constrain them to be equal across groups. Such choices are particularly relevant and various approaches exist to determine what item(s) should be used as anchor(s), both in MG-CCFA (French & Finch, 2008) and in MG-IRT (Candell & Drasgow, 1988; Wainer & Braun, 1988; Clauser et al., 1993; Khalid & Glas, 2014).

### Open practices:

The data and the analysis scripts are freely available and have been posted at https://osf.io/u9y8m/.

## Appendix

**Table 10:** Loadings’ convergence rate scale level - invariance scenario

			Convergence rate scale level - loadings		
N	C	J	MG-CCFA	MG-IRT LoR	MG-IRT LRT
250	**3**	**5**	0.996	0.992	1
		**25**	1	0.998	1
	**5**	**5**	1	1	1
		**25**	1	1	1
1000	**3**	**5**	1	1	1
		**25**	1	1	1
	**5**	**5**	1	1	1
		**25**	1	1	1

The bold entries were used to distinguish between design factors and do not refer to results

Note. MG-CCFA = Multiple-group categorical confirmatory factor analysis; MG-IRT LoR = Logistic regression with MG-IRT; MG-IRT LRT = Likelihood-ratio test with MG-IRT; *N* = Sample size within each group; C = Number of categories; J = Number of items

**Table 11:** Thresholds’ convergence rate scale level - invariance scenario

			Convergence rate scale level - thresholds		
N	C	J	MG-CCFA	MG-IRT LoR	MG-IRT LRT
250	**3**	**5**	0.996	0.998	1
		**25**	1	1	1
	**5**	**5**	0.998	1	1
		**25**	1	1	1
1000	**3**	**5**	1	1	1
		**25**	1	1	1
	**5**	**5**	1	1	1
		**25**	1	1	1

The bold entries were used to distinguish between design factors and do not refer to results

Note. MG-CCFA = Multiple-group categorical confirmatory factor analysis; MG-IRT LoR = Logistic regression with MG-IRT; MG-IRT LRT = Likelihood-ratio test with MG-IRT; N = Sample size within each group; C = Number of categories; J = Number of items

**Table 12:** Loadings’ convergence rate item level - invariance scenario

			Convergence rate item level - loadings		
N	C	J	MG-CCFA	MG-IRT LoR	MG-IRT LRT
250	**3**	**5**	0.998	0.996	1
		**25**	1	0.996	1
	**5**	**5**	1	1	1
		**25**	1	1	1
1000	**3**	**5**	1	1	1
		**25**	1	1	1
	**5**	**5**	1	1	1
		**25**	1	1	1

The bold entries were used to distinguish between design factors and do not refer to results

Note. MG-CCFA = Multiple-group categorical confirmatory factor analysis; MG-IRT LoR = Logistic regression with MG-IRT; MG-IRT LRT = Likelihood-ratio test with MG-IRT; *N* = Sample size within each group; C = Number of categories; J = Number of items

**Table 13:** Thresholds’ convergence rate item level - invariance scenario

			Convergence rate item level - thresholds		
N	C	J	MG-CCFA	MG-IRT LoR	MG-IRT LRT
250	**3**	**5**	0.996	0.998	1
		**25**	1	0.996	1
	**5**	**5**	1	1	1
		**25**	1	1	1
1000	**3**	**5**	1	1	1
		**25**	1	1	1
	**5**	**5**	1	1	1
		**25**	1	1	1

The bold entries were used to distinguish between design factors and do not refer to results

Note. MG-CCFA = Multiple-group categorical confirmatory factor analysis; MG-IRT LoR = Logistic regression with MG-IRT; MG-IRT LRT = Likelihood-ratio test with MG-IRT; *N* = Sample size within each group; C = Number of categories; J = Number of items

**Table 14:** Loadings’ convergence rate scale level - non-invariance scenario

				Convergence rate scale level - loadings					
				MG-CCFA		MG-IRT LoR		MG-IRT LRT	
N	C	J	%	small	large	small	large	small	large
250	**3**	**5**	**20%**	0.994	0.990	0.998	0.996	1	1
			**40%**	0.992	0.996	1	0.998	1	1
			**40% ±**	0.996	0.988	1	0.982	1	1
		**25**	**20%**	1	1	1	1	1	1
			**40%**	1	1	1	1	1	1
			**40% ±**	1	1	0.988	0.892	1	1
	**5**	**5**	**20%**	0.996	0.998	1	0.996	1	1
			**40%**	1	1	0.998	1	1	‘1
			**40% ±**	1	0.998	0.996	0.992	1	1
		**25**	**20%**	1	1	1	1	1	1
			**40%**	1	1	1	1	1	1
			**40% ±**	1	1	0.998	0.900	1	1
1000	**3**	**5**	**20%**	1	1	1	1	1	1
			**40%**	1	1	1	1	1	1
			**40% ±**	1	1	1	1	1	1
		**25**	**20%**	1	1	1	1	1	1
			**40%**	1	1	1	1	1	1
			**40% ±**	1	1	1	0.998	1	1
	**5**	**5**	**20%**	1	1	1	1	1	1
			**40%**	1	1	1	1	1	1
			**40% ±**	1	1	1	1	1	1
		**25**	**20%**	1	1	1	1	1	1
			**40%**	1	1	1	1	1	1
			**40% ±**	1	1	1	1	1	1

The bold entries were used to distinguish between design factors and do not refer to results

Note. MG-CCFA = Multiple-group categorical confirmatory factor analysis; MG-IRT LoR = Logistic regression with MG-IRT; MG-IRT LRT = Likelihood-ratio test with MG-IRT; *N* = Sample size within each group; C = Number of categories; J = Number of items; % = percentage of items affected by DIF (± misaligned); small = small bias; large = large bias

**Table 15:** Thresholds’ convergence rate scale level - non-invariance scenario

				Convergence rate scale level - thresholds					
				MG-CCFA		MG-IRT LoR		MG-IRT LRT	
N	C	J	%	small	large	small	large	small	large
250	**3**	**5**	**20%**	0.996	0.842	0.998	0.998	1	1
			**40%**	1	0.824	0.990	0.998	1	1
			**40% ±**	0.998	0.828	0.994	1	1	1
		**25**	**20%**	1	1	1	0.998	1	1
			**40%**	1	1	1	1	1	1
			**40% ±**	1	1	1	0.998	1	1
	**5**	**5**	**20%**	0.998	0.908	0.998	0.994	1	1
			**40%**	0.998	0.896	1	0.998	1	1
			**40% ±**	1	0.924	1	1	1	1
		**25**	**20%**	1	1	1	1	1	1
			**40%**	1	1	1	1	1	1
			**40% ±**	1	1	1	0.998	1	1
1000	**3**	**5**	**20%**	1	0.948	1	1	1	1
			**40%**	1	0.942	1	1	1	1
			**40% ±**	1	0.930	1	1	1	1
		**25**	**20%**	1	1	1	1	1	1
			**40%**	1	1	1	1	1	1
			**40% ±**	1	1	1	1	1	1
	**5**	**5**	**20%**	1	0.984	1	1	1	1
			**40%**	1	0.984	1	1	1	1
			**40% ±**	1	0.986	1	1	1	1
		**25**	**20%**	1	1	1	1	1	1
			**40%**	1	1	1	1	1	1
			**40% ±**	1	1	1	1	1	1

The bold entries were used to distinguish between design factors and do not refer to results

Note. MG-CCFA = Multiple-group categorical confirmatory factor analysis; MG-IRT LoR = Logistic regression with MG-IRT; MG-IRT LRT = Likelihood-ratio test with MG-IRT; *N* = Sample size within each group; C = Number of categories; J = Number of items; % = percentage of items affected by DIF (± misaligned); small = small bias; large = large bias

**Table 16:** Loadings’ convergence rate item level - non-invariance scenario

				Convergence rate item level - loadings					
				MG-CCFA		MG-IRT LoR		MG-IRT LRT	
N	C	J	%	small	large	small	large	small	large
250	**3**	**5**	**20%**	0.992	0.976	0.996	1	1	1
			**40%**	0.994	0.992	1	0.996	1	1
			**40% ±**	0.986	0.966	0.994	0.980	1	1
		**25**	**20%**	1	1	0.998	1	1	1
			**40%**	1	1	1	1	1	1
			**40% ±**	1	1	0.994	0.886	1	1
	**5**	**5**	**20%**	1	0.998	1	1	1	1
			**40%**	1	0.998	1	1	1	1
			**40% ±**	1	0.994	1	0.994	1	1
		**25**	**20%**	1	1	1	1	1	1
			**40%**	1	1	1	1	1	1
			**40% ±**	1	1	0.998	0.898	1	1
1000	**3**	**5**	**20%**	1	1	1	1	1	1
			**40%**	1	1	1	1	1	1
			**40% ±**	1	1	1	1	1	1
		**25**	**20%**	1	1	1	1	1	1
			**40%**	1	1	1	1	1	1
			**40% ±**	1	1	1	1	1	1
	**5**	**5**	**20%**	1	1	1	1	1	1
			**40%**	1	1	1	1	1	1
			**40% ±**	1	1	1	1	1	1
		**25**	**20%**	1	1	1	1	1	1
			**40%**	1	1	1	1	1	1
			**40% ±**	1	1	1	0.998	1	1

The bold entries were used to distinguish between design factors and do not refer to results

Note. MG-CCFA = Multiple-group categorical confirmatory factor analysis; MG-IRT LoR = Logistic regression with MG-IRT; MG-IRT LRT = Likelihood-ratio test with MG-IRT; *N* = Sample size within each group; C = Number of categories; J = Number of items; % = percentage of items affected by DIF (± misaligned); small = small bias; large = large bias

**Table 17:** Thresholds’ convergence rate item level - non-invariance scenario

				Convergence rate item level - thresholds					
				MG-CCFA		MG-IRT LoR		MG-IRT LRT	
N	C	J	%	small	large	small	large	small	large
250	**3**	**5**	**20%**	0.996	0.848	0.998	0.998	1	1
			**40%**	0.998	0.816	0.998	1	1	1
			**40% ±**	0.986	0.798	0.994	0.990	1	1
		**25**	**20%**	1	1	1	1	1	1
			**40%**	1	1	0.994	1	1	1
			**40% ±**	1	1	1	1	1	1
	**5**	**5**	**20%**	1	0.900	1	0.994	1	1
			**40%**	0.998	0.906	1	0.998	1	1
			**40% ±**	0.998	0.878	1	0.996	1	1
		**25**	**20%**	1	1	1	1	1	1
			**40%**	1	1	1	1	1	1
			**40% ±**	1	1	1	1	1	1
1000	**3**	**5**	**20%**	1	0.936	1	1	1	1
			**40%**	1	0.946	1	1	1	1
			**40% ±**	1	0.900	1	1	1	1
		**25**	**20%**	1	1	1	1	1	1
			**40%**	1	1	1	1	1	1
			**40% ±**	1	1	1	1	1	1
	**5**	**5**	**20%**	1	0.980	1	1	1	1
			**40%**	1	0.978	1	1	1	1
			**40% ±**	1	0.976	1	1	1	1
		**25**	**20%**	1	1	1	1	1	1
			**40%**	1	1	1	1	1	1
			**40% ±**	1	1	1	1	1	1

The bold entries were used to distinguish between design factors and do not refer to results

Note. MG-CCFA = Multiple-group categorical confirmatory factor analysis; MG-IRT LoR = Logistic regression with MG-IRT; MG-IRT LRT = Likelihood-ratio test with MG-IRT; *N* = Sample size within each group; C = Number of categories; J = Number of items; % = percentage of items affected by DIF (± misaligned); small = small bias; large = large bias

**Table 18:** Loadings’ FPR item level - non-invariance scenario

				FPR item level - loadings							
				MG-CCFA				MG-IRT			
				Comb		*χ* ^2^		LoR		LRT	
N	C	J	%	small	large	small	large	small	large	small	large
250	**3**	**5**	**20%**	0.042	0.044	0.049	0.052	0.059	0.048	0.045	0.051
			**40%**	0.053	0.050	0.058	0.058	0.053	0.049	0.049	0.037
			**40% ±**	0.046	0.066	0.051	0.067	0.060	0.077	0.056	0.051
		**25**	**20%**	0.002	0.001	0.063	0.070	0.002	0.002	0.051	0.050
			**40%**	0.003	0.001	0.071	0.111	0.001	0.002	0.047	0.056
			**40% ±**	0.001	0	0.054	0.055	0.001	0.001	0.045	0.047
	**5**	**5**	**20%**	0.051	0.044	0.060	0.049	0.005	0.008	0.045	0.056
			**40%**	0.062	0.069	0.067	0.074	0.005	0.005	0.065	0.059
			**40% ±**	0.047	0.072	0.051	0.072	0.010	0.007	0.046	0.044
		**25**	**20%**	0.002	0.001	0.064	0.074	0	0	0.050	0.047
			**40%**	0.004	0.001	0.080	0.127	0	0	0.048	0.046
			**40% ±**	0.001	0	0.056	0.059	0	0	0.050	0.048
1000	**3**	**5**	**20%**	0.039	0.038	0.062	0.063	0	0	0.047	0.053
			**40%**	0.038	0.064	0.072	0.129	0.001	0	0.058	0.064
			**40% ±**	0.030	0.033	0.063	0.104	0	0.003	0.058	0.043
		**25**	**20%**	0	0	0.063	0.105	0	0	0.055	0.051
			**40%**	0	0	0.108	0.262	0	0	0.045	0.043
			**40% ±**	0	0	0.052	0.053	0	0	0.057	0.044
	**5**	**5**	**20%**	0.027	0.029	0.051	0.045	0	0	0.064	0.046
			**40%**	0.043	0.059	0.079	0.137	0	0	0.049	0.062
			**40% ±**	0.045	0.029	0.075	0.133	0	0	0.048	0.056
		**25**	**20%**	0	0	0.067	0.121	0	0	0.044	0.053
			**40%**	0	0	0.125	0.328	0	0	0.046	0.051
			**40% ±**	0	0	0.049	0.055	0	0	0.049	0.053

The bold entries were used to distinguish between design factors and do not refer to results

Note. MG-CCFA = Multiple-group categorical confirmatory factor analysis; MG-IRT LoR = Logistic regression with MG-IRT; MG-IRT LRT = Likelihood-ratio test with MG-IRT; *N* = Sample size within each group; C = Number of categories; J = Number of items; % = percentage of items affected by DIF (± misaligned); small = small bias; large = large bias

**Table 19:** Thresholds’ FPR item level - non-invariance scenario

				FPR item level - thresholds							
				MG-CCFA				MG-IRT			
				Comb		*χ* ^2^		LoR		LRT	
N	C	J	%	small	large	small	large	small	large	small	large
250	**3**	**5**	**20%**	0.044	0.058	0.049	0.058	0.131	0.269	0.056	0.054
			**40%**	0.063	0.045	0.063	0.046	0.149	0.310	0.040	0.036
			**40% ±**	0.056	0.068	0.058	0.068	0.136	0.312	0.053	0.048
		**25**	**20%**	0	0	0.046	0.051	0.005	0.028	0.044	0.048
			**40%**	0	0	0.050	0.048	0.006	0.031	0.051	0.046
			**40% ±**	0	0	0.047	0.045	0.006	0.031	0.046	0.049
	**5**	**5**	**20%**	0.041	0.063	0.041	0.063	0.029	0.160	0.043	0.057
			**40%**	0.055	0.045	0.055	0.052	0.045	0.209	0.048	0.064
			**40% ±**	0.045	0.047	0.045	0.050	0.039	0.191	0.046	0.046
		**25**	**20%**	0	0	0.047	0.053	0	0.017	0.052	0.044
			**40%**	0	0	0.051	0.050	0.001	0.019	0.051	0.047
			**40% ±**	0	0	0.046	0.053	0	0.018	0.047	0.051
1000	**3**	**5**	**20%**	0.023	0.027	0.053	0.069	0.007	0.163	0.040	0.058
			**40%**	0.018	0.010	0.058	0.056	0.012	0.257	0.045	0.060
			**40% ±**	0.022	0.024	0.061	0.096	0.008	0.248	0.047	0.057
		**25**	**20%**	0	0	0.051	0.052	0	0.001	0.048	0.053
			**40%**	0	0	0.046	0.054	0	0	0.047	0.051
			**40% ±**	0	0	0.053	0.051	0	0.001	0.050	0.048
	**5**	**5**	**20%**	0.022	0.012	0.059	0.072	0	0.087	0.052	0.048
			**40%**	0.012	0.004	0.048	0.062	0	0.117	0.053	0.045
			**40% ±**	0.015	0.006	0.060	0.114	0	0.128	0.041	0.050
		**25**	**20%**	0	0	0.051	0.047	0	0	0.047	0.041
			**40%**	0	0	0.054	0.053	0	0	0.050	0.051
			**40% ±**	0	0	0.048	0.047	0	0	0.048	0.046

The bold entries were used to distinguish between design factors and do not refer to results

Note. MG-CCFA = Multiple-group categorical confirmatory factor analysis; MG-IRT LoR = Logistic regression with MG-IRT; MG-IRT LRT = Likelihood-ratio test with MG-IRT; *N* = Sample size within each group; C = Number of categories; J = Number of items; % = percentage of items affected by DIF (± misaligned); small = small bias; large = large bias

**Table 20:** Loadings’ TPR scale level - non-invariance scenario

				TPR scale level - loadings							
				MG-CCFA							
				Comb		*χ* ^2^		ΔRMSEA		ΔCFI	
N	C	J	%	small	large	small	large	small	large	small	large
250	**3**	**5**	**20%**	0.052	0.044	0.052	0.044	0.196	0.166	0.180	0.168
			**40%**	0.078	0.124	0.078	0.124	0.230	0.326	0.218	0.292
			**40% ±**	0.082	0.218	0.082	0.218	0.226	0.406	0.218	0.442
		**25**	**20%**	0.124	0.284	0.140	0.332	0.166	0.324	0.106	0.246
			**40%**	0.118	0.474	0.144	0.532	0.156	0.512	0.098	0.412
			**40% ±**	0.272	0.916	0.306	0.922	0.266	0.858	0.260	0.906
	**5**	**5**	**20%**	0.054	0.048	0.054	0.048	0.188	0.204	0.142	0.150
			**40%**	0.076	0.122	0.076	0.122	0.224	0.324	0.182	0.254
			**40% ±**	0.124	0.268	0.124	0.268	0.302	0.500	0.260	0.470
		**25**	**20%**	0.126	0.410	0.164	0.474	0.190	0.502	0.090	0.326
			**40%**	0.182	0.692	0.218	0.764	0.264	0.734	0.118	0.556
			**40% ±**	0.274	0.972	0.358	0.986	0.348	0.956	0.242	0.952
1000	**3**	**5**	**20%**	0.060	0.084	0.062	0.094	0.140	0.168	0.040	0.046
			**40%**	0.130	0.366	0.140	0.384	0.224	0.488	0.086	0.266
			**40% ±**	0.204	0.714	0.206	0.714	0.314	0.776	0.150	0.642
		**25**	**20%**	0.136	0.712	0.390	0.974	0.136	0.710	0	0.194
			**40%**	0.256	0.940	0.622	1	0.256	0.940	0.006	0.606
			**40% ±**	0.500	1	0.892	1	0.496	1	0.112	1
	**5**	**5**	**20%**	0.054	0.106	0.060	0.110	0.118	0.214	0.016	0.044
			**40%**	0.164	0.500	0.182	0.542	0.298	0.626	0.072	0.306
			**40% ±**	0.238	0.852	0.262	0.860	0.358	0.896	0.150	0.758
		**25**	**20%**	0.174	0.872	0.478	0.998	0.174	0.872	0.002	0.270
			**40%**	0.342	0.990	0.732	1	0.342	0.990	0.010	0.792
			**40% ±**	0.758	1	0.976	1	0.758	1	0.140	1

The bold entries were used to distinguish between design factors and do not refer to results

Note. MG-CCFA = Multiple-groups categorical confirmatory factor analysis; *N* = Number of Subjects within each group; C = Number of categories; J = Number of items; % = percentage of items affected by DIF (± misaligned); small = small bias; big = big bias

**Table 21:** Thresholds’ TPR scale level - non-invariance scenario

				TPR scale level - thresholds							
				MG-CCFA							
				Comb		*χ* ^2^		ΔRMSEA		ΔCFI	
N	C	J	%	small	large	small	large	small	large	small	large
250	**3**	**5**	**20%**	0.358	0.908	0.358	0.908	0.590	0.954	0.654	0.966
			**40%**	0.720	1	0.720	1	0.854	1	0.888	1
			**40% ±**	0.652	0.996	0.654	0.996	0.788	1	0.860	1
		**25**	**20%**	0.414	1	0.742	1	0.306	0.972	0.262	0.996
			**40%**	0.392	1	0.716	1	0.272	0.982	0.262	0.998
			**40% ±**	0.906	1	0.996	1	0.690	1	0.804	1
	**5**	**5**	**20%**	0.396	0.974	0.396	0.974	0.592	0.966	0.704	0.994
			**40%**	0.766	1	0.766	1	0.866	1	0.926	1
			**40% ±**	0.806	1	0.806	1	0.886	1	0.950	1
		**25**	**20%**	0.560	1	0.738	1	0.446	1	0.470	1
			**40%**	0.630	1	0.742	1	0.510	1	0.520	1
			**40% ±**	0.996	1	1	1	0.916	1	0.974	1
1000	**3**	**5**	**20%**	0.956	1	0.956	1	0.942	1	0.972	1
			**40%**	1	1	1	1	1	1	1	1
			**40% ±**	1	1	1	1	1	1	1	1
		**25**	**20%**	0.828	1	1	1	0.786	1	0.410	1
			**40%**	0.802	1	1	1	0.752	1	0.390	1
			**40% ±**	1	1	1	1	1	1	1	1
	**5**	**5**	**20%**	0.984	1	0.984	1	0.980	1	0.992	1
			**40%**	1	1	1	1	1	1	1	1
			**40% ±**	1	1	1	1	1	1	1	1
		**25**	**20%**	0.980	1	1	1	0.966	1	0.856	1
			**40%**	0.972	1	1	1	0.960	1	0.862	1
			**40% ±**	1	1	1	1	1	1	1	1

The bold entries were used to distinguish between design factors and do not refer to results

Note. MG-CCFA = Multiple-groups categorical confirmatory factor analysis; *N* = Number of Subjects within each group; C = Number of categories; J = Number of items; % = percentage of items affected by DIF (± misaligned); small = small bias; big = big bias

**Table 22:** Loadings’ TPR item level - non-invariance scenario

				TPR item level - loadings							
				MG-CCFA							
				Comb		*χ* ^2^		ΔRMSEA		ΔCFI	
N	C	J	%	small	large	small	large	small	large	small	large
250	**3**	**5**	**20%**	0.038	0.060	0.052	0.076	0.060	0.080	0.056	0.070
			**40%**	0.052	0.088	0.061	0.103	0.091	0.127	0.080	0.118
			**40% ±**	0.077	0.192	0.091	0.205	0.122	0.231	0.108	0.248
		**25**	**20%**	0.007	0.015	0.107	0.259	0.006	0.010	0.002	0.007
			**40%**	0.003	0.007	0.078	0.162	0.002	0.006	0	0.002
			**40% ±**	0.006	0.037	0.147	0.426	0.005	0.004	0.003	0.035
	**5**	**5**	**20%**	0.066	0.084	0.080	0.106	0.092	0.094	0.058	0.066
			**40%**	0.054	0.130	0.060	0.135	0.097	0.180	0.059	0.131
			**40% ±**	0.095	0.277	0.111	0.291	0.129	0.328	0.100	0.310
		**25**	**20%**	0.005	0.016	0.129	0.317	0.005	0.012	0.002	0.006
			**40%**	0.005	0.003	0.094	0.194	0.004	0.002	0.001	0.001
			**40% ±**	0.008	0.032	0.172	0.533	0.006	0.003	0.003	0.031
1000	**3**	**5**	**20%**	0.042	0.074	0.096	0.182	0.050	0.078	0.002	0.012
			**40%**	0.071	0.213	0.114	0.318	0.081	0.225	0.032	0.082
			**40% ±**	0.155	0.486	0.224	0.645	0.172	0.448	0.068	0.404
		**25**	**20%**	0	0.001	0.274	0.705	0	0.001	0	0
			**40%**	0	0	0.160	0.465	0	0	0	0
			**40% ±**	0	0.003	0.422	0.932	0	0	0	0.003
	**5**	**5**	**20%**	0.042	0.134	0.114	0.238	0.046	0.142	0.002	0.016
			**40%**	0.092	0.256	0.146	0.382	0.104	0.275	0.021	0.102
			**40% ±**	0.174	0.526	0.267	0.754	0.188	0.506	0.057	0.422
		**25**	**20%**	0.001	0	0.323	0.818	0.001	0	0	0
			**40%**	0	0	0.207	0.559	0	0	0	0
			**40% ±**	0	0.003	0.491	0.978	0	0	0	0.003

The bold entries were used to distinguish between design factors and do not refer to results

Note. MG-CCFA = Multiple-groups categorical confirmatory factor analysis; *N* = Number of Subjects within each group; C = Number of categories; J = Number of items; % = percentage of items affected by DIF (± misaligned); small = small bias; big = big bias

**Table 23:** Thresholds’ TPR item level - non-invariance scenario

				TPR item level - thresholds							
				MG-CCFA							
				Comb		*χ* ^2^		ΔRMSEA		ΔCFI	
N	C	J	%	small	large	small	large	small	large	small	large
250	**3**	**5**	**20%**	0.536	0.976	0.586	0.988	0.580	0.970	0.618	0.982
			**40%**	0.643	0.986	0.647	0.986	0.595	0.896	0.783	0.993
			**40% ±**	0.555	0.984	0.566	0.984	0.502	0.877	0.708	0.993
		**25**	**20%**	0.003	0.143	0.648	0.997	0.002	0.002	0	0.142
			**40%**	0.002	0.127	0.646	0.997	0.001	0	0.001	0.127
			**40% ±**	0	0.130	0.657	0.998	0	0	0	0.130
	**5**	**5**	**20%**	0.626	0.994	0.674	0.996	0.672	0.988	0.682	0.996
			**40%**	0.689	0.999	0.696	0.999	0.581	0.919	0.819	1
			**40% ±**	0.675	0.999	0.678	0.999	0.569	0.893	0.804	1
		**25**	**20%**	0.006	0.368	0.724	0.999	0.002	0	0.004	0.368
			**40%**	0.008	0.360	0.724	0.999	0.005	0	0.003	0.360
			**40% ±**	0.004	0.357	0.726	0.998	0	0	0.004	0.357
1000	**3**	**5**	**20%**	0.978	1	0.988	1	0.962	1	0.948	1
			**40%**	0.993	1	0.999	1	0.766	0.995	0.993	1
			**40% ±**	0.976	1	0.994	1	0.671	0.990	0.974	1
		**25**	**20%**	0	0.117	1	1	0	0	0	0.117
			**40%**	0	0.146	0.997	1	0	0	0	0.146
			**40% ±**	0	0.124	0.998	1	0	0	0	0.124
	**5**	**5**	**20%**	0.998	1	0.998	1	0.984	1	0.990	1
			**40%**	0.998	1	1	1	0.780	0.999	0.998	1
			**40% ±**	0.990	1	0.998	1	0.709	0.993	0.990	1
		**25**	**20%**	0	0.648	0.999	1	0	0	0	0.648
			**40%**	0	0.664	1	1	0	0	0	0.664
			**40% ±**	0	0.620	1	1	0	0	0	0.620

The bold entries were used to distinguish between design factors and do not refer to results

Note. MG-CCFA = Multiple-groups categorical confirmatory factor analysis; *N* = Number of Subjects within each group; C = Number of categories; J = Number of items; % = percentage of items affected by DIF (± misaligned); small = small bias; big = big bias

## Footnotes

1 Note that in our simulation studies, the length of the scale was varied only at two levels (5,25). For this reason, we advise the reader to be cautious in generalizing these results to scales of different lengths.
